# A national initiative in data science for health: an evaluation of the UK Farr Institute

**DOI:** 10.23889/ijpds.v5i1.1128

**Published:** 2020-04-08

**Authors:** H Hemingway, R Lyons, Q Li, I Buchan, J Ainsworth, J Pell, A Morris, Michael R Barnes, Helen Bedford, Marion Bennie, Ann Blandford, Andy Briggs, Peter Brocklehurst, Sinead Brophy, Gavin Brown, Paul Burton, Paul Burton, Christopher Butler, Simon Capewell, James Carpenter, John Carroll, Jackie A. Cassell, Fortunato Castillo, Mike Catchpole, Mark Caulfield, Helen Colhoun, Peter Coveney, Sarah Cunningham-Burley, Adnan Custovic, John Deanfield, Spiros Denaxas, Michael Dennis, Carol Dezateux, Chris Dibben, Peter Diggle, Will Dixon, Graham Dunn, Khaled El Emam, David Fone, David Ford, Ian Ford, John Frank, Nick Freemantle, Belinda Gabbe, John Gallacher, Martin Gibson, Ruth Gilbert, Mika Gissler, Carol Goble, Andy Goldberg, Mike Gravenor, David Gunnell, Phil Hannaford, Andrew Hayward, Matthew Hickman, Aroon Hingorani, Soren Holm, Cashel Holman, Gareth John, Ann John, Kerina Jones, Dipak Kalra, Graeme Laurie, Shon Lewis, Keith Lloyd, Sarah Lowe, Colin McCowan, John Macleod, Richard M. Martin, Anthony (Tony) Moore, Laurence Moore, Irwin Nazareth, Goran Nenadic, Shantini Paranjothy, Max Parmar, Richard Pebody, Steffen Petersen, Irene Petersen, Deenan Pillay, David Preen, Kate Pickett, Kathy Pritchard-Jones, Natasa Przulj, Andrew Renehan, Stephen Roberts, John Robson, Sarah Rodgers, Martin Rossor, Ian Russell, John Shawe-Taylor, Aziz Sheikh, Stefan Siebert, Helen Snooks, Matthew Sperrin, Judith Stephenson, Frank Sullivan, Chris Taylor, Paul Taylor, Adam Timmis, Hester J T Ward, John Williams, Paula Williamson, Alan Wilson, Olivia Wu, Mhairi Aitken, Ashley Akbari, Mohammad Al Sallakh, Sarah Al-Adely, Samantha Alvarez-Madrazo, Leslie Anne, Lorna Aucott, Rowena Bailey, Panos Balatsoukas, Amrita Bandyopadhyay, Mike Bartlett, Katherine Barutcu, Catherine Batchelder, Denise Beales, Miguel Belmonte, Brian Blower, Liam Brierley, Andrew Broadbent, Paul Burton, Mathilde Castagnet, Daniel Cave, Giovanna Ceroni, Tom Clemens, Huw Collins, Ed Conley, Carol-Ann Costello, Phil Couch, Lynsey Cross, James Cunningham, Marina Daskalopoulou, Emma Davies, Joanne Demmler, Emma Dixon, Christine Dobbs, Samuel Dredge, Vit Drga, Hannah Evans, Rachel Evans, Bassam Farran, Natalie Fitzpatrick, Michael Fleming, Beata Fonferko-Shadrach, Sarah Fox, Richard Fry, Catharine Goddard, Arturo Gonzalez-Izquierdo, Sharon Gordon, Ben Green, Benjamin Green, Nick Gresham, Lucy Griffiths, Rhiannon Griffiths, Rowena Griffiths, Kathy Haigh-Hutchinson, Melanie-Jayne Hainke, Phil Hannaford, Douglas Hardy, Steven Harris, Nina Hayes-Thompson, Martin Heaven, Neil Hillen, Kate Holmes, Elizabeth Irvine, Guy Jackson, Nadia Jackson, Emily Jefferson, David Jenkins, Camille Johnson, Jennifer Johnston, Michail Katsoulis, Daisy Kirkwood, Holger Kunz, Sabine Kurz, Arron Lacey, Amanda Lamb, Nathan Lea, Stephen Lloyd, Jane Lyons, Matt Machin, Noel Malod-Dognin, Louise Marryat, Cherry Martin, Gordon McAllister, Nicola McCleary, Lucy McCloughan, Paul McIntosh, Stephen Melia, Amy Mizen, Anna Mölter, Remi Momo, Alysha Morgan, Lynn Morrice, Kate Mortimer, Alireza Moayyeri, Chris Munro, Clifford Nangle, Thomas Nind, Ruth Norris, Laura North, Kieran O'Malley, Rhydian Owen, Adam Panagiotopoulos, Vaclav Papez, Richard Papworth, Laura Pasea, Steve Pavis, Jill Pell, Julia Petschnigg, Carol Porteous, Haider Raza, Emma Riordan, Heather Robinson, Nayha Sethi, Aneesha Singh, Claire Smith, Jiao Song, Markus Steiner, Paul Stephenson, Raj Tandon, Ed Tempest, Rachel Thompson, Sarah Toomey, Fatemeh Torabi, Leandro Tramma, Sandro Tsang, Wing-Chau Tung, Sam Turner, Victoria Turner, Sabine Van Der Veer, William Vance, Angharad Walters, Tony Whiffen, Andrew James Williams, Robert Aldridge, Samantha Alvarez-Madrazo, Athanasios Anastasiou, Amitava Banerjee, Damon Berridge, Corri Black, Paul Burton, Helen Colhoun, Sarah Cunningham-Burley, Juan-Pablo Casas Romero, Spiros Denaxas, Chris Dibben, Richard Dobson, Michael Fleming, Pia Hardelid, Katie Harron, Holger Kunz, Glen Martin, Georgina Moulton, Tanja Mueller, Niels Peek, Clifford Nangle, Nayha Sethi, Laura Shallcross, Matt Sperrin, Paul Taylor, Sabine van der Veer, Tjeerd van Staa, Alan Watkins, Elizabeth Williamson, Adeel Waheed, Nida Afzal, Harry Ahmed, Mohammad Annas Al Sallakh, Adel Alhlayl, Victoria Allan, Haitham Alzghaibi, Phil Appleby, Ellie Badrick, Kerry Bailey, Natalie Berry, Birgitta Bodegraven, Helen Brierley, Benjamin Brown, Giorgio Ciminata, Rosie Cornish, Eilidh Cowan, Mattea Deliu, Catherine Fitton, Michael Fleming, Kenny Haining, Kathryn Halliday, Elsie Horne, Rebecca Howard, Will Hulme, Myrto Kremyda-Vlachou, Emily Marchant, Glen Martin, Tanja Mueller, Jenny Newman, Julie Peconi, Tra My Pham, Rachel Reeves, Daniel Rhodes, William Rudgard, Catherine Smith, Georgious Spithourakis, Dennis Stallone Valentine, Grant Wyper, Bilal Yassine, Anna Zylbersztejn, Robert Aldridge, Amaya Azcoaga-Lorenzo, Ruth Blackburn, Ben Brown, Marco Caminati, Raymond Carragher, Sheng-Chia Chung, Caroline Dale, Spiros Denaxas, Chantal Edge, Ghazaleh Fatemifar, Mike Fleming, Julie George, Alison Hale, Michail Katsoulis, William Harvey, Lamiece Hassan, Watjana Lilaonitkul, Claudia Lindner, Serena Luchenski, Tom Lumbers, Claire Niedzwiedz, Meena Rafiq, Kristiina Rannikmae, Laura Shallcross, Smith Keith, Rona Strawbridge, Charlotte Warren-Gash, Honghan Wu, John Ainsworth, Dr Philip Couch, Professor David Ford, Simon Thompson, Dr Jacky Pallas, Professor Mark Parsons, Dr Emily Jefferson, Professor James Cunnigham, Georgina Moulton, Athanasios Anastasiou, Professor Paul Taylor, Dr Wing-Chau Tung, Dr Catharine Goddard, Professor Colin McCowan, Dr Shang-Ming Zhou, Innovative Governance, Dr Kerina Jones (Chair), Professor Graeme Laurie, Professor James Cunningham, Dr Nathan Lea, Harry Hemingway, Ms Rachel Evans, Professor David Ford, Ms Ruth Norris, Ms Natalie Fitzpatrick, Professor Sarah Cunningham-Burley (Chair), Dr Mhairi Aitken, Dr Chris Carrigan, Dr Lynsey Cross, Dr Simon Denegri, Ms Natalie Fitzpatrick, Dr Sarah Fox, Dr Lamiece Hassan, Ms Carol Porteous, Dr Mary Tully, Iain Buchan, Jeremy Wyatt, Ronan Lyons, Michaela Benzeval, Brendan Delaney, Frank Sullivan, Dr Alysha Morgan, Dr Rhydian Owen, Dr Denise Beales, Ms Nadia Jackson, Ms Daisy Kirkwood, Dr Wing-Chau Tung, Dr Catharine Goddard, Dr Lucy McCloughan, Ms Jennifer Johnston, Dr Amanda Lamb, Ms Ruth Norris, Ms Vicky Turner

**Affiliations:** Queen Mary University of London; University College London; University of Strathclyde; University College London; University of Glasgow; University College London (Currently Birmingham); Swansea University; University of Manchester; University of Leicester; University of Bristol; University; University of Liverpool; School of Hygiene and Tropical Medicine; University of Sussex; Brighton and Sussex Medical School; UCL Institute of Child Health; Public Health England; Queen Mary University of London; University of Dundee; University College London; University of Edinburgh; University of Manchester; University College London; University College London; Swansea University; University College London; University of Edinburgh; University of Liverpool/Lancaster; University of Manchester; University of Manchester; University of Ottawa; Cardiff University; Swansea University; University of Glasgow; Public Health Research and Policy, University of Edinburgh; University College London; Emergency and Trauma Research Unit in the Department of Epidemiology and Preventive Medicine, Monash University; University of Cardiff; University of Manchester; University College London; University of Manchester; University College London; Swansea University; University of Bristol; University of Aberdeen; University College London; University of Bristol; University College London; University of Manchester; University of Western Australia; NHS Wales Informatics Service; Swansea University; Swansea University; University College London; University of Edinburgh; University of Manchester; Swansea University; Welsh Government; University of Glasgow; University of Bristol; University of Bristol; University College London; University of Glasgow; University College London; University of Manchester; Cardiff University; University College London; Public Health England; Queen Mary University of London; University College London; University College London; University of Western Australia; University of York; University College London; University College London; University of Manchester; Swansea University; Queen Mary University of London; University of Liverpool; University College London; Swansea University; University College London; University of Edinburgh; Swansea University; Swansea University; University of Manchester; University College London; The University of St Andrews; University of Manchester; University College London; Queen Mary University of London; NHS National Services Scotland; Swansea University; University of Liverpool; University College London; University of Glasgow; University of Edinburgh; Swansea University; Swansea University; University of Manchester; University of Strathclyde; University of Edinburgh; University of Aberdeen; Swansea University; University of Manchester; Swansea University; University of Manchester; Swansea University; Swansea University; University College London; University of Manchester; University of Manchester; University of Edinburgh; University of Manchester; Newcastle University; Swansea University; University of Manchester; University College London; University of Edinburgh; Swansea University; University of Edinburgh; University of Manchester; University of Manchester; Swansea University; University of Manchester; University College London; University of Manchester; Swansea University; University of Manchester; Swansea University; Swansea University; University College London; University College London; University of Edinburgh; University of Edinburgh; University College London; University of Glasgow; Swansea University; University of Manchester; Swansea University; University of Dundee; University College London; University of Aberdeen; University of Manchester; University of Manchester; University of Manchester; Swansea University; Swansea University; Swansea University; University of Manchester; Swansea University; University of Aberdeen; University of Dundee; Swansea University; University of Manchester; Swansea University; University of Glasgow; University of Manchester; Swansea University; University of Manchester; University College London; University of Dundee; University of Manchester; University of Manchester; University of Dundee; University College London; University College London; University College London; University of Edinburgh; Swansea University; University of Manchester; University College London; University of Manchester; Swansea University; University of Manchester; University College London; University of Edinburgh; University of Edinburgh; University of Dundee; University of Edinburgh; University of Edinburgh; University of Dundee; University of Manchester; Swansea University; University of Manchester; University College London; Swansea University; University of Edinburgh; University of Manchester; University College London; University of Manchester; NHS National Services Scotland; University of Dundee; University of Manchester; Swansea University; University of Manchester; Swansea University; Edinburgh Law School; University College London; University of Glasgow; University College London; National Services Scotland; University of Glasgow; University College London; University College London; Swansea University; Swansea University; University of Manchester; Edinburgh Law School; University College London; University of Manchester; Swansea University; University of Aberdeen; University of Manchester; University of Manchester; University of Manchester; University of Manchester; Swansea University; Swansea University; University of Dundee; Swansea University; University College London; Swansea University; University of Manchester; University of Manchester; University of Manchester; Swansea University; Swansea University; Usher Institute for Population Health Sciences and Informatics, University of Edinburgh; University College London; University of Strathclyde; Swansea University Medical School, Swansea University; University College London; Swansea University; University of Aberdeen; Newcastle University; University of Edinburgh; Edinburgh Law School, University of Edinburgh; University College London; University College London; University of Edinburgh; University College London; University of Glasgow; University College London; University College London; University College London; University of Manchester; University of Manchester; University of Strathclyde; University of Manchester; University of Edinburgh; Edinburgh Law School; UCL Institute of Health Informatics, University College London; University of Manchester; University College London; University of Manchester; University of Manchester; Swansea University; London School of Hygiene & Tropical Medicine; Bradford Research Institute; University of Bradford; Cardiff University; Swansea University; Swansea University; University College London; Swansea University; University of Dundee; University of Manchester; Swansea University; University of Manchester; University of Manchester; University of Manchester; University of Manchester; University of Glasgow; University of Bristol; University of Edinburgh; University of Manchester; University of Aberdeen; University of Glasgow; University of Edinburgh; University of Edinburgh; University of Edinburgh; University of Manchester; University of Manchester; University College London; Swansea University; University of Manchester; University of Strathclyde; University of Liverpool; Swansea University; University College London; University College London; Queen Mary University of London; London School of Hygiene & Tropical Medicine; University College London; University College London; University College London; University of Strathclyde; University College London; University College London; University College London; University of St Andrews; University College London; University of Manchester; University of St Andrews; University of Strathclyde; University College London; University College London; University College London; University College London; University College London; University of Glasgow; University College London; Lancaster University; University College London; University of Glasgow; University of Manchester; University College London; University of Manchester; University College London; University College London; University of Glasgow; University College London; University of Edinburgh; University College London; University of Edinburgh; University of Glasgow; University College London; University of Edinburgh; University of Manchester; University of Manchester; Swansea University; Swansea University; University College London; University of Edinburgh; University of Dundee; Newcastle University; University of Manchester; Swansea University Medical School, Swansea University; University College London; Farr Institute of Health Informatics Research Network, University College London; Farr Institute of Health Informatics Research Network, University of Dundee; University of Glasgow; Swansea University; Swansea University; University of Edinburgh; Northumbria University; University College London; University College London; University of Edinburgh; Swansea University; University of Manchester; University College London; University of Edinburgh; University of Edinburgh; University of Leeds; Swansea University; University College London; University College London; University of Manchester; University of Manchester; University of Edinburgh; University of Manchester; University of Liverpool; University of Birmingham; Swansea University; University of Essex; Imperial College London; The University of St Andrews; Swansea University; Swansea University; University College London; University College London; University College London; University College London; University of Dundee; University of Edinburgh; University of Dundee; University of Manchester; University of Manchester; University of Manchester; 1 HDR UK London; 2 UCL Institute of Health Informatics, 222 Euston Road, London NW1 2DA; 3 HDRUK Wales/Northern Ireland; 4 Swansea University Medical School, Fourth Floor, Data Science Building, Singleton Campus, Swansea, SA2 8PP; 5 West China Hospital, Chengdu, China; 6 University of Liverpool, Liverpool L69 3BX; 7 Division of Informatics, Imaging & Data Sciences, The University of Manchester, Oxford Rd, Manchester M13 9PL; 8 Institute of Health and Wellbeing, University of Glasgow, 1 Lilybank Gardens, Glasgow G12 8RZ; 9 University of Edinburgh; 10 HDR UK

## Abstract

**Objective:**

To evaluate the extent to which the inter-institutional, inter-disciplinary mobilisation of data and skills in the Farr Institute contributed to establishing the emerging field of data science for health in the UK.

**Design and Outcome measures:**

We evaluated evidence of six domains characterising a new field of science:

- defining central scientific challenges,
- demonstrating how the central challenges might be solved,
- creating novel interactions among groups of scientists,
- training new types of experts,
- re-organising universities,
- demonstrating impacts in society.

We carried out citation, network and time trend analyses of publications, and a narrative review of infrastructure, methods and tools.

**Setting:**

Four UK centres in London, North England, Scotland and Wales (23 university partners), 2013-2018.

**Results:**

1. The Farr Institute helped define a central scientific challenge publishing a research corpus, demonstrating insights from electronic health record (EHR) and administrative data at each stage of the translational cycle in 593 papers with at least one Farr Institute author affiliation on PubMed. 2. The Farr Institute offered some demonstrations of how these scientific challenges might be solved: it established the first four ISO27001 certified trusted research environments in the UK, and approved more than 1000 research users, published on 102 unique EHR and administrative data sources, although there was no clear evidence of an increase in novel, sustained record linkages. The Farr Institute established open platforms for the EHR phenotyping algorithms and validations (>70 diseases, CALIBER). Sample sizes showed some evidence of increase but remained less than 10% of the UK population in primary care-hospital care linked studies. 3.The Farr Institute created novel interactions among researchers: the co-author publication network expanded from 944 unique co-authors (based on 67 publications in the first 30 months) to 3839 unique co-authors (545 papers in the final 30 months). 4. Training expanded substantially with 3 new masters courses, training >400 people at masters, short-course and leadership level and 48 PhD students. 5. Universities reorganised with 4/5 Centres established 27 new faculty (tenured) positions, 3 new university institutes. 6. Emerging evidence of impacts included: > 3200 citations for the 10 most cited papers and Farr research informed eight practice-changing clinical guidelines and policies relevant to the health of millions of UK citizens.

**Conclusion:**

The Farr Institute played a major role in establishing and growing the field of data science for health in the UK, with some initial evidence of benefits for health and healthcare. The Farr Institute has now expanded into Health Data Research (HDR) UK but key challenges remain including, how to network such activities internationally.

## What is already known

- National research initiatives in data science for health are underway in several countries seeking to harness insights from electronic health record (EHR) and administrative data at regional and national scale for patient and public benefit.
- One approach to grow this emerging field, adopted by the UK, was to establish a dedicated national research institute, the Farr Institute.
- We do not know how effective such initiatives are. Multi-centre, inter-disciplinary research initiatives are common, but there is a lack of research evaluating such initiatives particularly national research institutes.
- The Farr Institute ran from 2013 until 2018 when its larger-scale successor, Health Data Research (HDR) UK, was established.

## What this study adds

- We provide a framework of six domains relevant for evaluating new inter-institutional, inter-disciplinary initiatives seeking to establish and grow an emerging field of science: defining central scientific challenges, demonstrating how the central challenges might be solved, creating novel interactions among groups of scientists, training new types of experts, re-organising universities, demonstrating impacts in society.
- We show that the Farr Institute created new activities in and across each of the six domains for developing a distinctive research field.
- The Farr Institute demonstrated the ability of multiple UK health research funders and multiple universities to partner in mobilising data, methodology and expertise across disciplines, organisations and information governance domains, resulting in a larger scale of research and improved methodology.
- We have demonstrated globally relevant challenges and opportunities for developing data science for health across instutional and disciplinary barriers, consistent with the need for big investigation not simply big data.
- First, there is a need for a framework by which HDR UK and other national research initiatives might be more rigorously evaluated.

## Introduction

### Rationale for national initiatives in data science for health

Countries across the globe are increasing efforts to harness research insights from EHR and administrative data. A common theme across these initiatives is to access data for research on a bigger scale (number of subjects included in analyses) and with greater detail of clinical and related information, in order to advance a wide range of research: from disease causation and classification to drug discovery, translational research, clinical trials, evidence-based medicine, clinical practice and public health. While many fields of science have few relevant national borders, data science for health has inherent national and regional dimensions: regional clinical knowledge is required to understand the context and validity of data, and countries differ in the legal, political, economic and public opinion context shaping the research using such data. Countries differ in their approach, for example: nationwide administrative and registry data in Nordic countries [1–4]; province –wide initiatives in Canada [5] and Australia [6], networks of hospitals in the German Medical Informatics Initiative [7] and US PCORnet [8]; and genomics/precision medicine initiatives linked to EHRs such as the US Million Veteran Program [9] and *All of Us* [10].

### UK establishes national research institute

The UK decided a national research institute was necessary to develop and deliver data science for health in order to leverage the NHS and allied data sources. The UK has a population of 65 million, a single payer health system (one NHS), a unique identifier for its citizens’ health data, long-standing population-wide EHRs, and 2.2 million citizens in consented cohorts, many with genomic and other detailed research data [11], and an ambition to have 5 million NHS patients with sequenced genomes [12].

**Figure 1: UK map of four Farr Institute Centres and partners (N=23):**
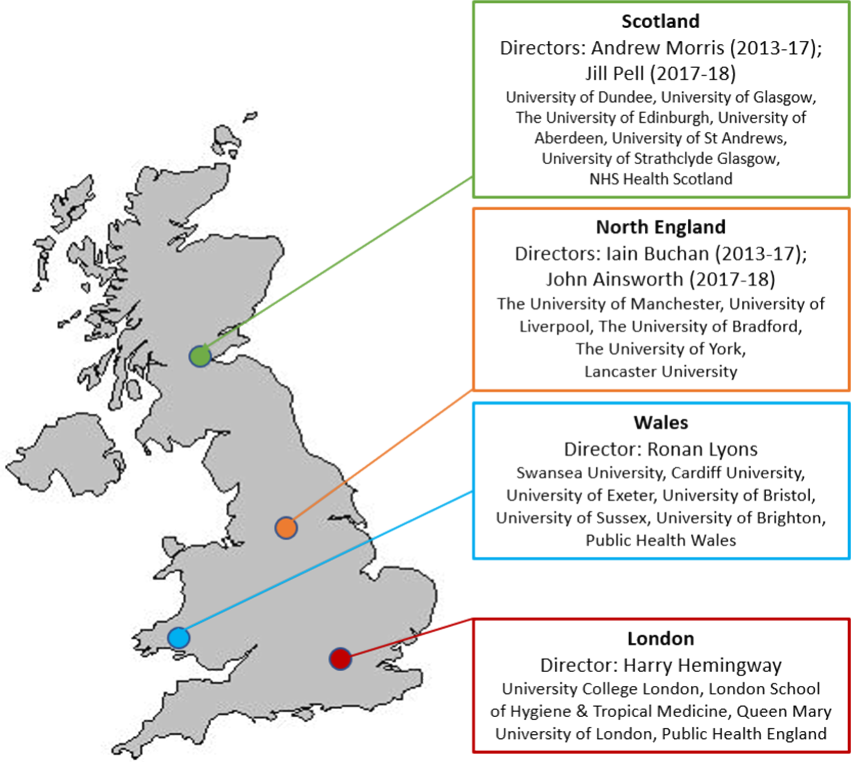


The Farr Institute was established in 2013 (supplementary Box 1) to leverage these assets and build capabilities to do research that could only, or best, be done at national scale. The funding for the Farr Institute came from a consortium of ten government and charitable research funders, awarding four academic centres (Scotland, Wales, Northern England and London), involving partnerships across 21 universities in the four centres (Figure 1). In 2018 the Farr Institute made way for the expanded successor institute, HDR UK, with new centres and longer-term core funding (a comparison of the Farr Institute and HDR UK is shown in Table 1).

### Objectives

Empirical evaluations of inter-institutional, inter-disciplinary research initiatives, or indeed national research institutes, have seldom been reported [13, 14]. Evaluations may be conflicted by an interest in persuading funders to continue support. The Farr Institute does not have such a conflict and thus provides an opportunity to learn from its five years of experience. The Farr Institute was a national experiment in how to build the foundations for a new research field of data science for health at scale. In the absence of a well-established framework for evaluating national research institutes, we based our evaluation on the domains of a new field of science, following previous literature [15] and the initial strategy [16] of a research institute in a different field of science, but also launching in 2013 (the Francis Crick Institute).

## Methods

We evaluated six inter-related domains of a new field of science (see Box), and for each domain sought evidence (publicly accessible and peer reviewed where available) of change during the five years of the Farr Institute (April 2013-March 2018). The evaluation was retrospective and was not planned in 2013.

**Box: Domains for evaluating new field of science and sources of evidence used:** 

Domain	Source of information
Defining central scientific challenges	Strategy documents
	Themes, scale, sources, linkages, from 100 most significant publications
Demonstrating how the central challenges might be solved	Survey of Farr Centres: infrastructure, new data made accessible, EHR phenotyping
Novel interactions among groups of scientists	Co-author publication networks, based on all publications with Farr affiliation n=3200
Training new types of experts	Survey of Farr Centres
Re-organising Universities	Survey of Farr Centres
Demonstrating impacts in society	Clinical guidelines and policy documents citing Farr Institute research, changing practice.

### Defining central scientific challenges

In order to evaluate how the Farr Institute defined central scientific challenges we analysed published strategy documents and annual reports (available on the Farr Institute website) and invited each Centre to nominate their most scientifically significant publications (25 per centre, 100 total). We (QL and HH) extracted information from these 100 full text publications on attribution to the Farr Institute, different science themes, disease area, scale, number of data sources, new record linkages, and evidence of science impact from citation tracking (Google Scholar, accessed September 2019).

To be eligible, each publication reported the use of one or more source of EHR or administrative data or methods directly relevant to data science. Consented studies without use of such EHR or administrative data were not eligible. We classified research themes (following strategy documents) as: citizen driven health, discovery science, quality and outcomes, trials and public health. We defined attribution to the Farr Institute as at least one author who: listed Farr Institute as an author affiliation in PubMed, acknowledged funding for the Farr Institute, or was in receipt of funding from the Farr Institute. Data sources were classified as primary care, hospital discharge data (e.g., Hospital Episode Statistics for England, Patient Episode Database for Wales, Scottish Morbidity Record), detailed hospital data, disease and procedure registries, mortality, other health, and socio-economic and other non-health data. We extracted for each publication the number of people providing the denominator population (sample size) and classified the population as healthy (general population sample) or based on specific disease or procedure. We carried out a structured survey with each Centre completing information on how the central challenges might be solved in three areas: e-infrastructure and platforms for accessing and sharing data, new data sources made available for researchers, and EHR phenotyping methods for structured and unstructured data.

For novel interactions among groups of scientists we analysed peer reviewed publications. In addition to the in-depth analysis of the self nominated 100 most significant publications, we also sought to identify all publications with at least one author listing a Farr affiliation. We searched PubMed from inception to 18 September 2018, using the strategy ‘Farr Institute[AD]’. We visualised the change over time in scientist network behaviour in the Farr Institute, with co-author publications networks based on all publications returned from PubMed, using Cytoscape cross-institution activity by year of publication. We analysed co-author publication network and inter-disciplinarity by the halfway time point, comparing the first vs the final 30 months of the Farr Institute.

For training new types of experts and Re-organising Universities, we carried out a structured survey across centres.

For impact in society, we identified clinical guidelines and policy documents citing Farr Institute publications through annual reports to funders, automated software used by funders to capture a range of outputs and impacts (Researchfish) and by contacting investigators.

## Results

### Defining central scientific challenges

**Figure 2: Central scientific challenges of the Farr Institute:**
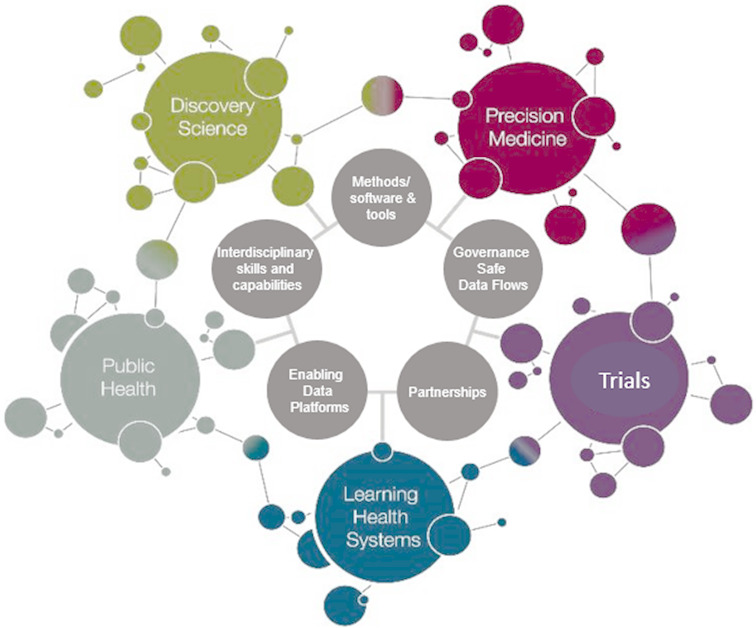


The Farr Institute published a research corpus around a central scientific challenge, demonstrating insights from EHRs and administrative data. Figure 2 (top panel) illustrates the challenges of the Farr Institute and (middle panel) demonstrates the corresponding research publication corpus. The proportion (%) of publications at different stages of the translational cycle was: methods (17%), patient involvement (3%), discovery science (8%), health services research (24%), clinical trials (5%) and public health (42%). The Farr Institute published highly-cited papers using EHR at each stage in the translational cycle (the top 10 are shown in Table 2), with a total of 3200 citations. Four of these highly-cited papers illustrate the higher resolution of using linked EHR [17–20]. These different research themes were applied across different clinical areas, including cardiometabolic, maternity and child health, mental health, cancer, renal and respiratory.

**Figure 3: Sample size as a percentage of total UK population reported in research publications:**
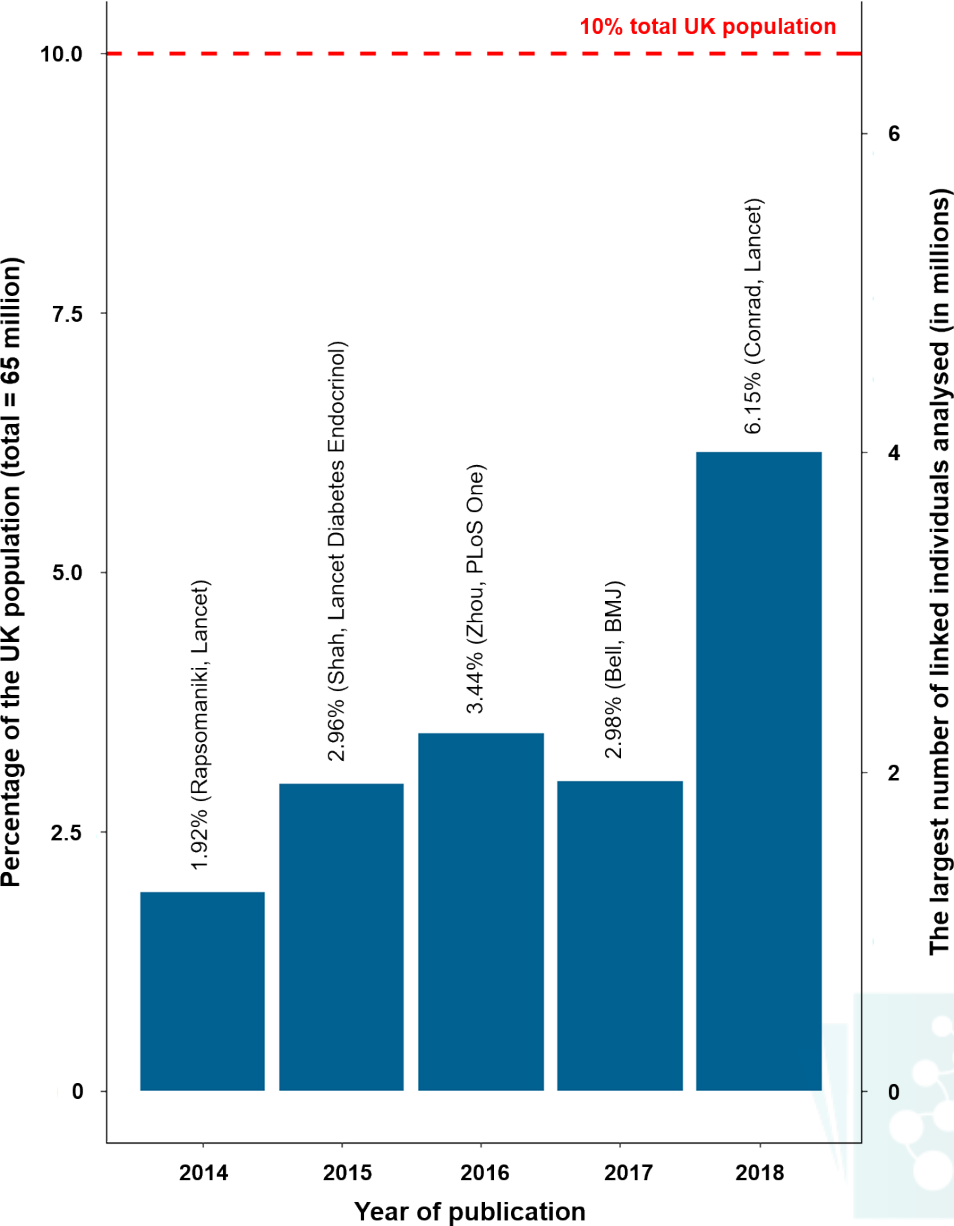


There was some evidence of a modest increase in the scale (number of people analysed in the denominator population) of research over time in these publications (Figure 3), based on linked primary-secondary care data in adults. But by 2018 this represented only 6.15% of the UK population [21]. There was just one paper that used the whole of England’s hospitalisation data: Freemantle and colleagues [22] analysing weekend mortality effects using 14.8 million admissions, and several using all England’s deaths data.

### Demonstrating how the central challenges might be solved: access to research-ready data

In 2013 there were no independently accredited Trusted Research Environments (TRE) for NHS data: by 2018 there were four (one in each centre) ISO 27001:2013 certified data safe havens (Table 3a). The TREs provide secure remote access, a safe environment for the analysis of sensitive patient identifiable data, a pre-requisite for receiving unconsented, individual level health data for research use. We found evidence that Farr activity enabled other scientific fields: with over 1000 approved users on these four data safe havens working on over 300 research projects (the majority being external, having no Farr Institute funding). The Farr Institute enabled the research use of diverse anonymised patient records, linked across primary care and secondary care, including NHS imaging data, blood laboratory values and reimbursed prescriptions (Table 3b). There was a cumulative total of 102 unique data sources reported in these publications (Figure 4a), with 13% from primary care, 17% limited coded hospital data, 8% detailed hospital data, 19% registries of disease and procedures, 26% socio-economic and environment, 6% death data and 12% other health data. The setting and names of each unique data source reported in these publications are shown in Supplementary Table 1. We found that the median number of record sources per publication showed no evidence of increasing, and if anything decreased over the 5 years (Figure 4b).

**Figure 4a: Cumulative total of unique electronic health record data sources reported in Farr Institute publications:**
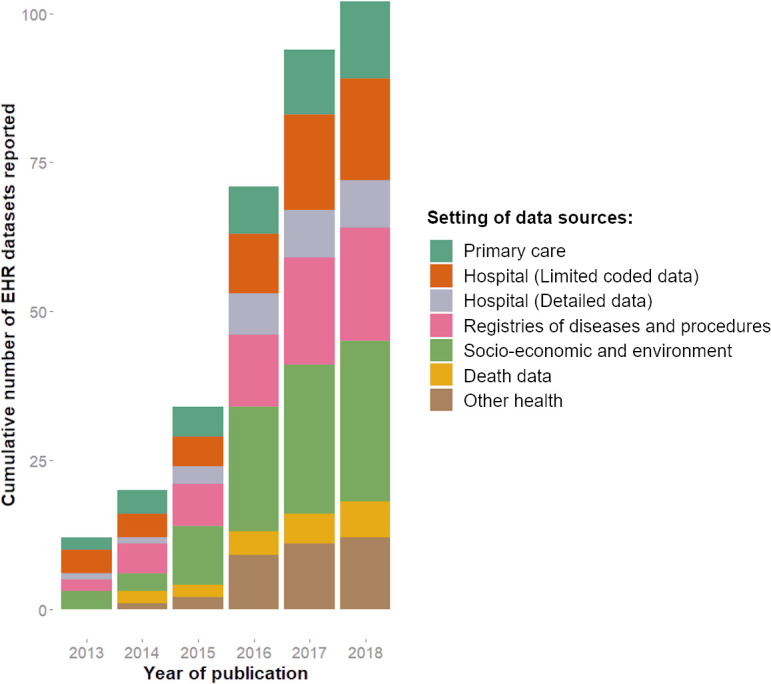


**Figure 4b: Number of electronic health record data sources reported in each publication (from Farr top 100):**
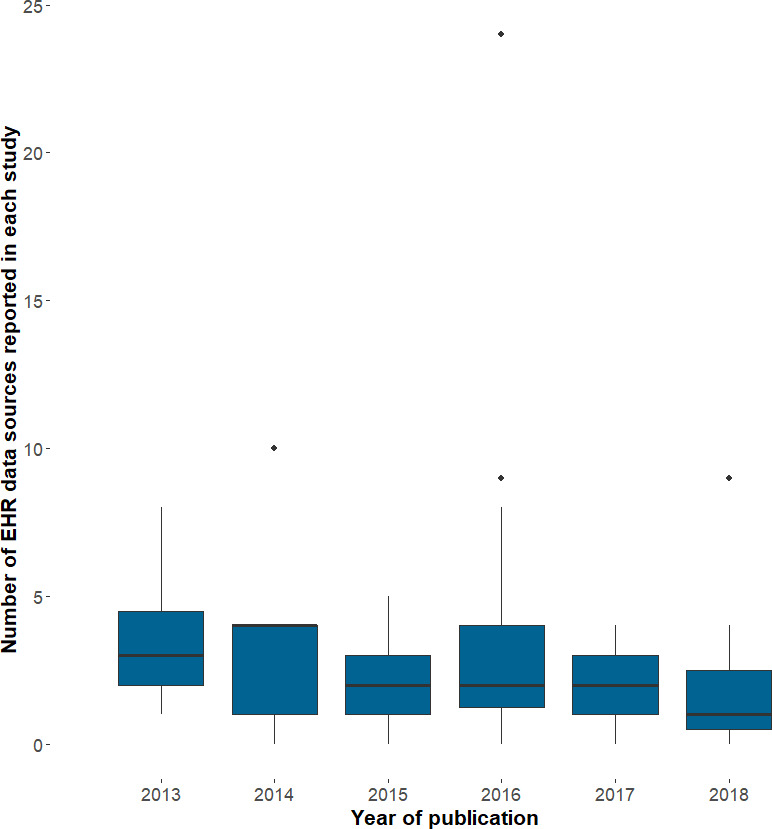


### Demonstrating how the central challenges might be solved: phenotyping

In 2013 there were no openly accessible portals for defining diseases and health-related conditions using EHR data (EHR ‘phenotyping’). The Farr Institute supported several initiatives in disease phenotyping (Table 3c): these included CALIBER, an open platform [23] of re-usable EHR phenotypes (code lists + logic + validations) for over 70 diseases which have been re-used in more than 50 publications with more than 80 ongoing projects [24]. In addition, there were several publications of EHR phenotypes in Wales and Scotland [25] and a clinical code repository [26].

Methods of surfacing the entire structured and unstructured data in a hospital have now been demonstrated in three hospitals with CogStack and SemEHR [27]. 

### Creating Novel interactions among groups of scientists

Based on author affiliation, the search [Farr Institute[AD]] on PubMed returned 594 unique publications (from inception to 18 September 2018). Figure 5 shows that there was a large expansion of co-author publication networks comparing the first 30 months (67 publications with 944 unique co-authors) and the final 30 months (545 papers and 3839 unique co-authors). SupplementaryFigure 4 shows that overall across the 100 publications, 28% included Farr Institute as both author affiliation and funder acknowledgement, 14% as author affiliation only, 11% as funder acknowledgement only and 42% as Farr-funded investigator only, as confirmed by the centres. There was some evidence that over time both author affiliation and funder acknowledgement increased. Based on the departmental affiliation of co-authors there was some evidence of greater inter-disciplinarity in the last 30 months of the Farr Institute compared to the initial 30 months (Supplementary Figure 5). We identified 17% of publications involving universities from across two or more Farr Centres.

**Figure 5: Change in co-author publication networks between the first and final 30-month periods (each dot represents a unique author: lines connect authors publishing together):**
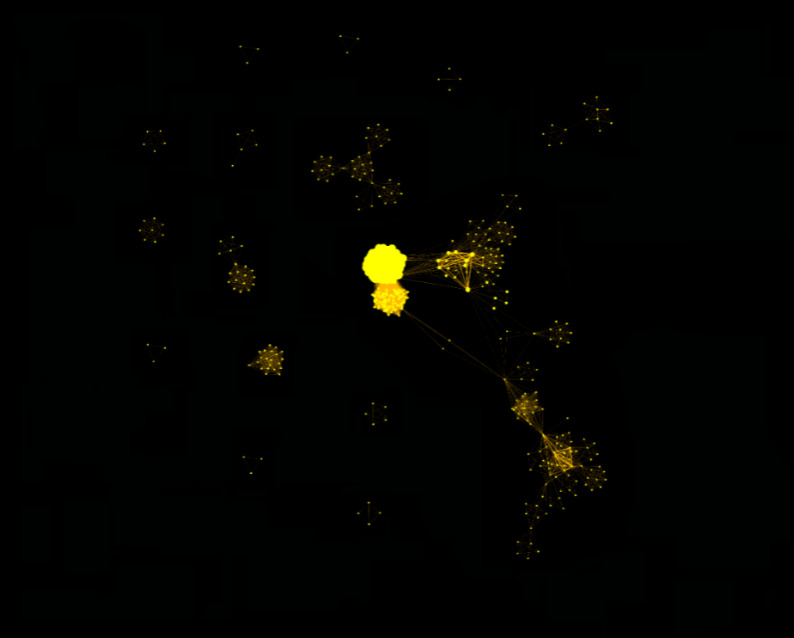

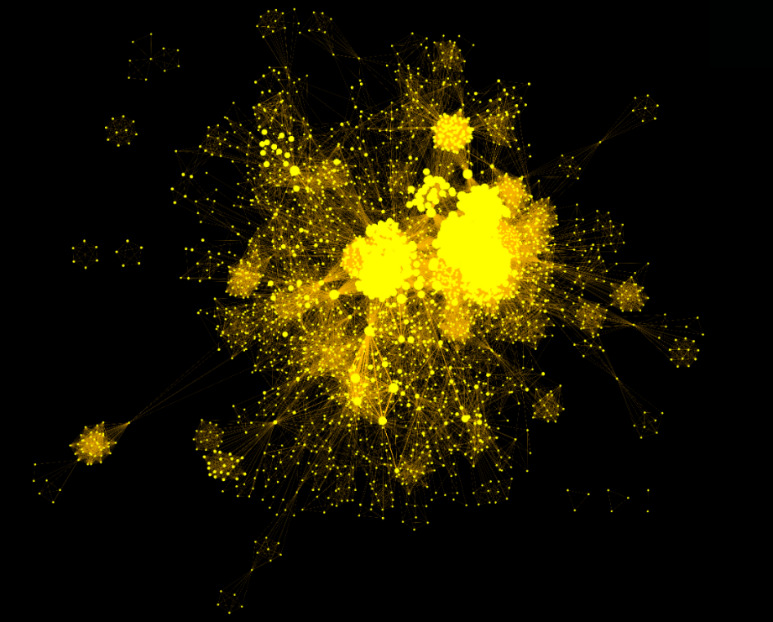
a) First 30 months

b) final 30 months

We explored the number of publications among the top 100 papers with an international (non-UK) author affiliation and found the total number of publications as follows: 17 publications non-Nordic Europe, 11 US, 10 Nordic, 8 Canada, 4 Australia and 2 China.

Among the 100 papers identified by the Farr Institute, 87 were found in the Scopus database, which provides structured institution data used to generate Supplementary Figure 6. The median (IQR) number of universities per Farr Institute publication was 3 (IQR: 2-4) in 2013 and 6 (IQR: 4-8) in 2018 (Supplementary Figure 6). In April 2017 the Farr Institute with the European Federation of Medical Informatics attracted over 850 delegates from around the world to Informatics for Health [28] – the UK’s largest health and biomedical informatics research gathering to date.

### Training new types of experts

The Farr Institute Centres created eight new training programmes at masters, short-course and leadership level across the four Farr Centres (Table 4). In total 2102 people were trained in informatics and directly relevant courses across the 4 Centres. As well as conventional programmes - there were 48 PhD students directly funded by the Farr Institute – the Farr Institute also established a competitively awarded Farr Future Leaders residential programme with two cohorts of 21 mid-career researchers with facilitation of national research collaborations in conjunction with leadership training.

### Re-organising Universities

Universities associated with the Farr Institute established new institutes and structures (cost centres) for health informatics and data science. At UCL (Institute of Health Informatics, 2014), University of Manchester (Division of Informatics, Imaging and Data Sciences, Health eResearch Centre), Edinburgh (Usher Institute of Population Sciences and Informatics), Swansea University (Patient and Population Health Informatics). These universities established 24 Faculty (tenured) posts as enduring leverage of the Farr award. 

### Demonstrating impacts in society

Farr Institute research at different stages of the translational cycle was cited in and informed practice-changing clinical guidelines and policies Table 5. These examples included research in public health (WHO guideline recommendations on video observed therapy for TB), clinical risk prediction (European Society of Cardiology), drug regulatory approvals (NICE potentially affecting the type or duration of drug treatment), and implementation of genomic medicine. The change in practice recommendations potentially affects more than a million UK citizens.

More widely Farr informatics research informed changes in government strategy from a centralised to a decentralised approach to integrating place-based health and administrative data for multiple analytic purposes [29]. This work also generated a £20m pilot of problem-based data integration, pulling through data by addressing care pathway blockages and research questions of importance to the local community in regions of 3-5m population [30]. This became the blueprint for England’s Local Health and Care Record Exemplars [31].

## Discussion

Clinicians, patients and policy makers have growing expectations of the use of data to provide research insights with the potential to improve health and care outcomes [32]. New scientific fields tend to have high-priority defining characteristics; we provide evidence in six recognized domains suggesting the Farr Institute played a major role in establishing and growing the field of data science for health in the UK. The experience of the Farr Institute has informed the design of Health Data Research UK (HDR UK), and this evaluation is relevant to the inter-institution, inter-disciplinary challenges of scaling up health science around big data in many parts of the world.

### Evolution of UK national research institute in policy context

A substantial achievement of the Farr Institute and its funders was the founding of its larger successor HDR UK. The key differences and similarities of the two organisations are shown in Table 1. This expansion from Farr Institute to HDR UK was recommended by the Farr Institute International Advisory Board in 2015, and much of the evolution of the Farr (see timeline in Supplementary Fig 3) was in preparation for this change. Lessons learned from the Farr Institute informed the development of HDR UK’s science strategy [33] (with 6 themes in human phenome project, AI, multiomics and multi-disease aetiology, clinical trials, digital health insights, and public health) and delivery, establishing a new single legal entity, single Director and Board, working via consortium agreements. The expanded research organisation partnerships in HDR UK now includes the majority of UK research organisations with significant expertise in data science for health. During 2013-18 the UK underwent a set of profound political, societal and policy changes relevant to data science for health: implemented the most significant reforms of the NHS of a generation in 2013 (establishing NHS Digital, Public Health England), enduring multiple crises of public trust over the use of data (care.data, Royal Free NHS Foundation Trust and Google DeepMind), the most significant changes in data protection legislation in a generation (as a result of the EU General Data Protection Regulation), two General Elections, and the historic referendum to leave the EU (see timeline in Supplementary Fig 4).

### International context of national research initiatives in data science for health

The Farr Institute and HDR UK have sought to learn from the differing approaches among countries and jurisdictions to advancing data science for health. For example, in order to facilitate learning across national boundaries, the Farr Institute jointly funded exchange fellowships with the Institute of Clinical Evaluative Sciences in Ontario, Canada. Currently, as far as the authors are aware, other countries have not established a national research institute dedicated to data science for health directly analogous to the Farr Institute or HDR UK. There is a need for strategic collaboration across national borders in data science for health [34], with proposals for working across national borders in Nordic countries [4].

The UK, informed by the ability of smaller countries such as Denmark [35] to deliver nationwide research built on an array of linkable record resources, as established the national institute in order to meet the challenging goal of nationwide research in a country of 65 million people. Some of the challenges facing the Farr Institute, and now HDR UK, are common to any research initiative based on catalysing inter-institutional and inter-disciplinary collaboration. Previous policy reports have recommended the need for intra-national methodological developments in data science for health as an important basis for international collaboration [36]. 

### Defining central scientific challenges: biomedical

The central scientific challenge set out by the 2011 funding competition (supplementary MRC 2011 Funding call) concerned record linkages. After the Farr Institute was established, the international advisory board, funders and directors recognised the need to more clearly identify the key research challenges which could only or best be addressed with centres coming together as a single national research institute. This led, in 2015, to the development of the science themes of discovery science, precision medicine, trials, learning health systems and public health outlined in Figure 2.

### Defining central scientific challenges: scale

Providing a ‘more powerful telescope’ by enabling EHR and administrative data at greater scale (larger sample sizes) is part of the central scientific challenge. Although nationwide primary care data exist in the UK with the 65 million population held by four GP system suppliers, they are not brought together in a single dataset for research purposes. The Farr Institute was publishing on 6% samples at 2018. The largest collections are held by Q-Research, Clinical Practice Research Datalink (CPRD) and System One, and there are more extensive collections of linked GP and other care data in the regions and devolved administrations, such as the Secure Anonymised Information Linkage (SAIL) system in Wales and the Connected Health Cities’ Trustworthy Research Environments in North England’s four regions. The levers for increasing scale do not sit in research institutes, but with data controllers and the legal and political environments. The Farr Institute paved the way for federation of research data queries and distributed analytics across regional data aggregations.

### Defining central scientific challenges: across the translational cycle

Most biomedical research disciplines are focused on a particular phase of the translational cycle: the Farr Institute demonstrated that a distinctive contribution of data science for health is that EHR and other sources of data ‘in the wild’ can link investigators across all phases of the translational research cycle. The Farr Institute made a start in the UK: the ambition, which HDR UK has taken on, is to constructively disrupt current models of evidence-based medicine, clinical practice and translational research, including the way that research is organised and funded.

### Defining central scientific challenges: record linkages

The original 2011 funding competition (supplementary MRC funding call document) sought centres to “undertake and promote innovative linkage and analysis of large health related data sets”. The top 100 Farr Institute publications reported 102 unique data sets, but record linkages were not reported consistently, nor in sufficient detail to know which sources had been linked, and whether any linkage was new. In Wales (SAIL), Scotland (eIDRIS) and some English regions (Connected Health Cities) there are data linkage and trustworthy research environments that have fuelled numerous research outputs. For example, in Wales primary care data (including text) are linked to hospital admissions data, dispensed prescribing, blood laboratory values and a wide range of socio-economic data. This breadth and depth of linkages, and their sustained accessibility by researchers, have not emerged across larger populations such as England. In England the opportunities for developing a growing, sustainable environment for record linkages were severely curtailed by care.data and have only recovered in the regional devolved approaches such as the NHS England Local Health and Care Record Exemplars. In annotating Farr Institute research publications, we found variable clarity on reporting of record linkage and were not able to easily identify how many linkages had been reported which were new and which might be readily accessible to future researchers.

### Defining central scientific challenges: data quality

Improvements in the quality of health record data, an internationally recognised challenge [35], was not an aim of the original funding competition, nor did it become a co-ordinated national focus in the Farr Institute, in part because the key influences lie in the NHS. Nonetheless the Farr Institute did deliver an open portal, CALIBER, for EHR phenotyping algorithms, along with evaluations of data quality and validations.

### Demonstrating how the central challenges might be solved

The Farr Institute transformed the UK’s ability to bring non-consented individual-level health data into trustworthy environments and make them available for other researchers, based on specifically approved projects. Nonetheless, there remain many different data governance environments and processes for data access for research, with much room for harmonisation and streamlining. We demonstrate here how the Farr Institute published on over 100 EHR and administrative data sources; in some situations these were the first research use of these data. Despite the undoubted progress reported here, the EHR data sources reported represent a tiny proportion of available data.

### How the challenge may be addressed: phenotyping

The Farr Institute demonstrated approaches to the major challenge of defining disease and health related traits with diverse EHR and other data. The development of the open CALIBER portal for EHR phenotype algorithms and their validations provides a basis on which to develop a national online facility to integrate data, methods and investigators for EHR phenotyping. Recent work from the Farr Institute demonstrated multiple code set validations of the 308 most common diseases and conditions, providing a ‘chronological map’ of human health from birth to death [37,38]. A key challenge for the future is to develop approaches which provide a degree of consistency and replicability across jurisdictions and national borders. 

### Creating novel interactions among groups of scientists

We visualised an ‘explosion’ of co-author networks. This reflects the willingness of investigators to self-identify with the Farr Institute, as there was no monitoring of this practice at central or national level, as well as extensive collaborations between those with and without Farr Institute funding. The top ten most cited Farr publications have been cited in total >3200 times. The capital investment allowed the co-ordinated establishment, for the first time in the UK, of physical homes (buildings) for the emerging field: 6421m^2^ of dedicated, branded space allowing research disciplines, technical specialists, NHS IT professionals and industry partners to ‘breathe the same air’. Beyond 2018, the capital investment in the Farr Institute has had an enduring effect, with most of the physical estate now being dedicated to HDR UK activities. The Farr Institute co-sponsored substantial expansion of scientific conferences: >850 delegates from 13 countries at Manchester 2017 meeting (>3 times larger than 2013 conference). Two new journals were established (*International Journal of Population Data Science* and *Journal of Learning Health Systems*) with Farr investigators Kerina Jones and Iain Buchan as editors.

There remain major challenges in bringing scientists and technical specialists to work together across different institutions and disciplines. Indeed the influential Research Excellence Framework, provides financial incentives to universities to compete, rather than collaborate [39]. 

### Impact on Innovation and Industry Partnerships

Universities within the Farr Institute Centres collaborated with industrial partners on research (e.g. Astra Zeneca, see Table 5), and training (e.g. masters courses with an option for students to complete their dissertation as interns in industry). However with 21 university partners the Farr Institute was not able to sign strategic industry partnerships. 

### Training new types of experts

At masters, short-course and leadership levels, the Farr Institute had a substantial effect on teaching and training. The Farr Institute evolved its training opportunities in response to the growing need for data science as a discipline and in leadership, neither of which were explicitly envisaged in the 2011 original funding call (supplementary MRC 2011 Funding call). 

### Demonstrating impacts in society: on health and healthcare

The Farr Institute carried out research underpinning policies and recommendations to change clinical and public health practice, and shaping government policy in health data management and digital health innovation. We provide here examples of specific research findings and their relation to changes in policy and recommendations. However, the Farr Institute had no central mechanism of identifying such influence (Table 5 is likely incomplete), nor of prospectively following research through policy recommendations to measure changes in health. In some cases Farr research may have impacts in later years; HDR UK might usefully establish a more systematic approach.

### Demonstrating impacts in society: public engagement and trust

The Farr Institute directly funded the involvement and engagement of the public and patients in research using patient data. In 2013 there were no national campaigns involving patients and the public in research on patient data. By 2018, the Farr Institute had delivered influential national campaigns in public engagement with the ‘100 ways’ case studies, explaining to patients and public examples of the benefits of Farr Institute research (13,000 followers and subscribers), and the #datasaveslives campaign, which has generated more than 50,000 retweets. In addition, the Farr Institute contributed to the Understanding Patient Data Wellcome Trust initiative [40]. The Farr Institute established public panels, a network of over 50 regular public contributors, and using a range of deliberative methods engaged members of the public in dialogue around the ways that data are used in health research; consulting them on research and governance practices and involving them in agenda-setting and decision-making processes.

### Limitations and Challenges

We identify five important limitations to this evaluation and briefly discuss how these limitations might be addressed for others evaluating inter-institutional, inter-disciplinary research initiatives. First, the challenge of attribution: would the changes in each domain which we catalogue here have happened anyway, in the absence of the Farr Institute? Because the funding of the Farr was intended to catalyse and leverage a range of activities, rather than to wholly fund specific activities, it is seldom possible to prove attribution robustly.This challenge of attribution is illustrated with research publications. In nearly all research publications analysed in this evaluation, the Farr Institute was one of several funders, and co-authors with Farr funding are usually among a larger group of co-authors without Farr funding. The Farr Institute did not have an intra-mural programme of research fully funded by the Farr awards, which were initially made to build four centres. Unsurprisingly therefore, the major peer-reviewed scientific outputs of the Farr were not published by all four centres together. This illustrates the challenge of transparent and publicly accessible attribution to a national institute.

Second, the Farr Institute did not design a prospective evaluation at the outset and thus the evidence presented here has inherent limitations of a post hoc evaluation. A key lesson learned was the importance of establishing at the outset national data systems allowing research about research ideally in near real time. These key data elements include items that currently do have unique identifiers: Farr investigators (identified by ORCID iDs), their publications (identified by PMID), but also the Farr Institute could have done more to advance central cataloguing (with unique identifiers) of datasets, record linkages, projects, disciplines, employing organisation and department, partner organisations (e.g. NHS). Indeed, much of the evidence reported in this evaluation could not have been identified by others, and some of it remains not open to independent scrutiny.

Third, evaluations require comparisons and ours was limited to the first and final 30 months (a ‘before and after’ design). We believe it would be informative in future to compare with other inter-institutional, inter-disciplinary national research institutes (including for example in the UK, the Crick, Turing, Sanger, Dementia Research Institute).

Fourth, our evaluation was neither external to nor independent of, the Farr Institute. However, the Farr Institute did receive regular external feedback, from both the International Advisory Board and the funder consortium. Since the Farr Institute ended in 2018, there were no potential conflicts of trying to seek further funding.

Fifth, we recognize that there is a role for qualitative evidence, for example through interviews with stakeholders as well as critics, which might add insights and highlight further critical challenges on many of the domains which we sought to evaluate, including the nature, and extent, of any changes attributable to the Farr Institute.

## Conclusion

In the UK, the Farr Institute played a significant role in beginning to grow the field of data science for health. In 2013 there was little UK-wide co-ordination or visibility of research capabilities, including methods development or training in data science for health, and by 2018 this had been transformed. The importance of a national research institute in this field is evidenced by the UK’s expanded commitment to HDR UK.

## Ethics statement

Ethical committee approval was not required for this evaluation.

## Supplementary Material

Supplementary Appendix
