# Supplementary material for: A national initiative in data science for health: an evaluation of the UK Farr Institute
Source: Int J Popul Data Sci. 2020 Apr 8;5(1):1128. doi: 10.23889/ijpds.v5i1.1128 (PMC7480324; doi:10.23889/ijpds.v5i1.1128)
Supplement: Supplementary Appendix [file ijpds-05-1128-s001.pdf]

Table 1: Comparison of the UK Farr Insitute and Health Data Research UK

	**Farr Institute (past)**	**HDR UK (present)**
**Years of operation**	**2013-2018**	**2018 + (15-year horizon)**
**Purpose**	Galvanise UK research community<br>With initial focus on record linkage	Directed science<br>Bigger ambition:<br>Data science for health and healthcare: 6 themes:<br>https://www.hdruk.ac.uk/research/
**Cross-centre peer reviewed science?**	No	Yes. Activity matched funding to require cross Site science
**Inter-disciplinarily**	Funding call focused on record linkage, epidemiology + health informatics	Greater inter-disciplinarily: more emphasis on understanding disease mechanism (e.g. with multi-omics), and more computer science /AI
**Funders**	10 including MRC, EPSRC, ESRC, NIHR, WT, BHF, CRUK, Arthritis Research UK, Scottish and Welsh Office.	9 as before + Northern Ireland (not CRUK, ARUK)
**Initial funding**	£18m revenue (+£20m one off capital in 2013 + £1m network)	£110m revenue
**Structure and Governance**	Loose academic coalition<br>4 Directors: no formal Board or Executive	Company limited by guarantee<br>1 Director, Board, Executive
	Centres (lead university)<br>**London (part)**<br>• University College London<br>**Scotland**<br>• University of Dundee<br>**Wales**<br>• Swansea University<br>**Manchester**<br>• University of Manchester<br>(full partners listed on map, Figure 1)	**Sites** (co-ordinating research organisation) &<br>**Pan-London** (University College London)<br>• Imperial, King's and University College London<br>• London School of Hygiene & Tropical Medicine<br>• Queen Mary University London<br>**Scotland** (University of Edinburgh)<br>• University of Aberdeen, Dundee, Edinburgh, Glasgow, St Andrews & Strathclyde<br>**Wales/Northern Ireland** (Swansea University)<br>• Queen's University Belfast<br>• Swansea University<br>**Midlands** (University of Birmingham)<br>• University Hospitals Birmingham NHS Foundation Trust<br>• University of Birmingham, Leicester, Nottingham and Warwick<br>**University of Oxford** (no partners)<br>**Cambridge** (WT Sanger Institute)<br>• Cambridge University Hospitals NHS Foundation Trust<br>• EMBL-European Bioinformatics Institute<br>• University of Cambridge<br>• Wellcome Trust Sanger Institute
**Contractual agreements between Centres / Sites**	None	Institute Agreement signed by co-ordinating research organisation in each Site and HDR UK
**Contractual agreements between co-ordinating research organisation and local partners**	None	Institute Agreement signed by co-ordinating research organisation in each Site and HDR UK
**Location of central team**	Edinburgh	London

Table 2. Central scientific challenge: EHR research at each stage of the translational cycle, illustrated by the 10 most highly cited research publications

Theme	Advance	Potential impact	Reference	Citations (Google Scholar)
Benefits of scale: High resolution epidemiology	Association of BMI with wide range of incidence cancers. Not previously reported at this scale (N=5m, primary care EHR not linked to HES data)	Prevention of cancer	Bhaskaran et al., Lancet 2014[17]	806
Benefits of scale: High resolution epidemiology	Association of blood pressure with wide range of incident cardiovascular diseases, beyond heart attack and stroke. Risk of 12 cardiovascular diseases not previously reported . N=1.9m, primary care data linked to HES CALIBER platform	Choice of endpoint in trials	Rapsomaniki et al., Lancet 2014[19]	695
Methods: transparent reporting	Reporting studies conducted using observational routinely collected health data (RECORD statement)	Reproducibility in science	Benchimol et al., PLoS Med 2015[41]	
Benefits of scale: High resolution epidemiology	Association of diabetes with wide range of incident cardiovascular diseases beyond heart attack and stroke. Risk of 12 cardiovascular diseases not previously reported . N=1.9m, primary care data linked to HES CALIBER platform	Choice of endpoints in trials	Shah et al., Lancet Diabetes Endocrinol, 2015[42]	447
Nationwide evaluation of care and outcomes	International comparisons of whole health system data on acute myocardial infarction	NHS care	Chung et al., Lancet 2014[18]	220
Benefits of linkage: Methods	Linking 4 national structured data sources (CALIBER) for ascertaining and sub-typing acute myocardial infarction: demonstrating quality of coding.	Quality of data	Herrett et al., BMJ 2013[43]	227
Vaccine effectiveness	Zoster vaccine effectiveness against incident zoster and, importantly, post-herpetic neuralgia	Disease prevention	Langan et al., PLoS Med 2013[44]	175
Primary care trials at point of care	Point of care randomised trials:<br>2 exemplars e Lung and RETROPRO	Capacity to mount trials	vanStaa et al., HTA 2014[45]	115
Healthcare	7 day working week and mortality using HES data and stroke registry	Junior doctors contracts	Freemantle et al., BMJ 2015[22] and Bray et al., Lancet 2016[46]	242
Genetic Discovery	Human knockouts First study of human knockouts among parentally related adults.<br>First English study reporting linkage of primary care health records to genomic samples	Efficiency in drug development	Narasimhan et al., Science 2016[47]	136

Citation assessment performed 18th September 2019. EHR = Electronic Health Records, HES = Health Episode Statistics.

Table 3a . How this challenge is addressed: new national eInfrastructure platforms for accessing and sharing data

Infrastructure/challenge	What was achieved?	References	What was the contribution of Farr	Evidence of impact and change 2013-2018
UK SECURE Eresearch PLATFORM (UKSeRP) **UK has >100 consented cohorts but had no platform for distributed team science to access multiple cohorts**	53 cohorts engaged covering 2m people: data from 21 cohorts covering 687,000 people available on website. The MRC's Dementias Research Platform UK (DPUK) was the first major tenant with multiple UK cohorts). Other cohorts utilising UKSeRP are: UK Biobank (access to distributed outcome adjudication team), ALSPAC, AirWave and East London Genes for Health.	UKSeRP: Jones et al., Lancet 2016 [48] DPUK: https://portal.dementiasplatform.uk/[49]	Farr researchers created this key piece of national eInfrastructure.	50 applications for data; 25 approved and 11 underway. DPUK design informed similar models in US, Canada, Australia, South Korea. Platform extending research to other disease areas, e.g. stroke https://www.dementiasplatform.uk/news/uk-wide-research-programme-to-investigate-dementia-in-stroke-patients[50] UKSeRP a key component to providing access to routine data and images in multiple bids in Life Sciences Industrial Strategy.
TRUSTED RESEARCH ENVIRONMENTS (TRE) **How to establish secure data analytics facilities for identifiable patient data: allowing safe data, safe people, safe platforms**	For the first time in the UK created ISO27001 accredited or Scottish Government accredited Safe Havens. 4 created: one per centre: SAIL HeRC TRE National Data Safe Haven (Scotland) CALIBER, London	www.saildatabank.com [51] Jones et al., Journal of Biomedical Informatics 2014 [52] www.herc.ac.uk/tre [53] www.isdscotland.org/products-and-services/eDRIS [54]	Farr researchers designed, built and operate the TREs. Financial resources for office accommodation and HPC computer; creation of a multidisciplinary environment (academic, informatics and NHS Staff). SAIL now a tenant on the robust UKSeRP infrastructure.	Our trusted research environments count a total of 2780 CPU cores and 3.0PB of storage, and a combined user base of in excess of 1000 researchers and in excess of 300 research projects completed or in flight. HeRC TRE provides capability for GM Connected Health City (www.connectedhealthcities.org) [55], Manchester BRC, DPUK sensing platform, MRC Clinical Proteomics Centre. Scotland: as whole, 131 live projects are underway, with access to records of 5.3 million people, and more than 200 dataset linkages made. Similar design underway in India and Brazil. Informed design of Digital Innovation Hubs in Life Sciences Industrial Strategy 2017.
CLOUD COMPUTE **Multi-dimensional data requires scalable storage and compute capability eMEDLAB MRC CLIMB**	Shared infrastructure in off-site datacentre with over 9,000 cores and 4Pb data. UCL, QMUL, LSHTM, Crick, Sanger, EBI, KCL and Farr London and Genomics England Award winning, at the time one of the largest private biomedical clouds in Europe.	http://www.emedlab.ac.uk/ [56] https://www.climb.ac.uk/ [57]	Farr researchers were core to the design and development of eMEDLAB, but it was funded by separate MRC award. The CLIMB infrastructure was designed and is managed by Farr investigator (Thompson).	eMedLab is a joint project with 6 institutions - UCL, QMUL, Crick, Sanger, KCL, EBI. The CLIMB project (Cloud Infrastructure for Microbial Bioinformatics) is a collaboration between Warwick, Birmingham, Cardiff, Swansea, Bath and Leicester Universities and The Quadram Institute Bioscience to develop and deploy a world leading cyber-infrastructure for microbial bioinformatics; providing free cloud-based compute, storage, and analysis tools for academic microbiologists in the UK.
JISC SAFE SHARE **How to authorise researchers to remotely access and share data safely and securely, from their own project site – with information governance rules, varying with sensitivity of the data**	A federated identity management system and a high assurance network overlay encrypted to National Cyber Security Centre (NCSC) standards.	https://www.jisc.ac.uk/safe-share [58]	Farr researchers initiated the project with JISC and were involved in all stages of the project from requirements to evaluation of the service.	From the success of the Safe Share pilot project, JISC have added Safe Share to their service catalogue, providing service to ADRN and PSN users.

*Table 3b. How this challenge is addressed: Data made accessible in trusted research environments (data safe havens)**

**Datasets***	**Linked primary-secondary care data**	**What was achieved?**	**Data safe haven and access procedures**
**Scotland NHS Data**<br>1.4m hospital admissions per year, 90m community prescriptions per year, plus other hospital activity data<br>Acute admissions (SMR 01), Psychiatric admissions, (SMR 04), Maternity (SMR02), Child health surveillance, Community prescribing, NRS vital events (such as births, deaths, still births, etc.), Cancer registry (SMR06), Diabetes (SCI-DC)	No	450 live projects, with 130 accessing data. Multiple publications based on Scottish hospital data.<br>NHS data taken into safe haven and prepared for research use. Quicker provisioning of data.	National Safe Haven and eDRIS National network of accredited federated safe havens<br><br>http://www.isdscotland.org/Products-and-Services/eDRIS/[54]
**Scotland NHS data: imaging**: a copy of all (1.5 m) historical images from the clinical NPACS into the Scottish Medical Imaging Database (SMI) on to the computing hardware at EPCC. ~1.5 million cases with an estimated total size of 81TB have been transferred.	No	An architectural solution and *Irdmp prototype* for handling big imaging data within a safe haven were designed and implemented. Currently being extended to run on the National Safe Haven hosted by Edinburgh Performance Computing Centre (EPCC) and to provide an anonymised extract from SMI for a small exemplar research project.	To date ~1.5 million cases with an estimated total size of 81TB have been transferred. Solution has been used to provide data for 4 different consented research projects linking phenotypic data with routinely collected imaging data within the SH in HIC.
**Welsh NHS Data**<br>Includes: primary care, acute admissions, laboratory results, psychiatric admissions, maternity, Child health surveillance, Community prescribing, vital status (such as births, deaths, still births, etc.), cancer registry, wider societal data	Yes	Farr helped enhance SAIL with additional data sources, including to 86M rows of laboratory results, hospital dispensing data and fields derived from NLP of free text from clinical correspondence.	www.saildatabank.com[51]<br><br>https://saildatabank.com/the-sail-databank-10-years-of-spearheading-data-privacy-and-research-utility/
**English NHS Data**<br>Linking primary care (CPRD), hospital episode statistics, disease registry (acute myocardial infarction) and death registry with phenotyping and other tools on CALIBER platform	Yes	Farr delivered the secure remote access and developed the platform of methods and tools: used in over 50 publications by 20 research groups; internationally presented at NIH workshop on data science.	UCL Data safehaven<br>https://www.ucl.ac.uk/health-informatics/caliber[23]
**Regional English NHS Data: detailed in-hospital data e.g. NIHR Health Informatics Collaborative Critical Care**<br>UCLH, King's Imperial, Oxford, and Cambridge hospitals<br>40,000 unique admissions and 120 million data items	No	Farr provided Data Safe Haven, informatics expertise (Denaxas); first ever data sharing agreements and infrastructure to share data across 5 English NHS trusts.<br><br>Datathon with US open data in critical care (MIMIC) Aboab et al., Science Translational Medicine 2016. [59]	UCL Data Safe haven<br><br>Harris et al., Int J Med Inform 2018[60]
**Regional English NHS Data: Greater Manchester HeRC Data Well Information Exchange:** linked primary secondary and social care data with capacity for linking to mobile and wearables	Yes	Coverage of a population of up to 2.8 million people	HeRC Trusted Research Environment

Note: * A complete list of EHR and administrative resources reported in Farr publications is provided in Supplementary Table 1

Table 3c. How this challenge is addressed: EHR Phenotyping methods with structured and unstructured EHR data and wearables

Challenge	What was achieved?	Reference	Farr contribution	Evidence of impact
**Structured data: defining EHR disease phenotypes** EHR Phenotyping which are described by multiple EHR codes across primary and secondary care poses challenges for replication and scale	Developed an open platform of methods and tools (e.g. with semantic web technologies) for computable, reusable phenotypes of in primary care and secondary care data. Web-based repository of code lists, linked to publications, available to download. Software for the CALIBER research platform including tools to manage codelists and prepare electronic health record data for analysis	www.caliberresearch.com[23] > papers www.clinicalcodes.org[26] Springate et al., PLOS ONE 2014[61]	Dr Denaxas and Dr Shah have been a core members of the Farr team since 2013	Internationally one of the largest open resources generating a 'library' of EHR disease phenotypes with >70 complex diseases phenotyped papers using these tools highly cited
Identifying disease phenotypes From high dimensional EHR codes	Treated disease phenotying as a problem of text categorization proposed a machine learning driven framework to automatically identify useful signals that define the condition.	Zhou et al, PLoS One[25]	Zhou and colleagues were members of the Farr CIPHER team	The framework being applied to defining different conditions from EHRs and extended to unstructured data
Information about the same condition may be held in multiple EHR sources	Linked 4 national structured data sources for ascertaining and sub-typing acute myocardial infarction<br>Linked data sources from England, Wales and Scotland<br>Includes extensive published code, algorithm, synthetic data github.com/RenalHDRUK	Herrett et al., BMJ 2013[43]<br>Sawhney et al., AdvChronicKidney Dis 2017[62]<br>Sawhney et al., BMJ open 2018[63]	Black, Smeeth, Chess, Peek core members of Farr team since 2013; Project fully funded by Farr in 2017	Approach to definition and validation (clinical expert review, risk factors, prognostic and genetic) informing approaches in UK Biobank
Unstructured data mapping text to SNOMED-CT	National Research Data Appliances deployed in all health boards and trusts in Wales to facilitate data management, linkage and facilities for analysing free-text data using GATE software	Fonferko-Shadrach et al., Seizure 2017[64]	Simon Thompson David Ford / Ronan Lyons	System now being extended to local authorities for ingestion of social services and other databases
**Searching the entire hospital record** structured and unstructured, in near real-time for alerting, research, trials and audit/coding	CogStack&SemEHR<br>Open source information retrieval and extraction platform implementing best-of-breed enterprise search, natural language processing (including semantic annotation), analytics and visualization technologies to unlock the health record and assist in clinical decision making and research.	Jackson et al., BMC Med InformDecis Mak 2018[65]<br><br>Wu et al., JAMIA 2018[66]	Richard Dobson has been a member of the Farr team since 2015 (jointly with KCL)	Implemented in 3 London hospitals. Highlighted as key enabling infrastructure in CMO Generation Genome report<br>Lasting impact on direct patient care through real time alerting, patient recruitment (eg to Genomics England, streamlining services (eg patient flow) and research through optional de-id step
**Prescribing:** to allow 'synchronous' prescribing	Free text matching Algorithm<br>A natural language processing algorithm for converting text drug doses to a set of structured fields (dose amount, units, frequency etc.) [1], which is published as an open source R package (http://caliberanalysis.r-forge.r-project.org/)	published as an open source R package (http://caliberanalysis.r-forge.r-project.org/)[23]	Dr Shah has been a core member of the Farr team since 2013.	4.6 million patients in Vision systems.<br>Being implemented in clinical practice for synchronizing prescriptions (Vision)
**Prescribing:** to ACC coding	Natural language processing to transform drug dosage instructions into quantification of drug exposure over time and connection to Anatomical Chemical Classification coding. The Prescribing Information System (PIS) holds over 1.6 billion prescriptions reimbursed in the community from January 1993 to 2014, with linkage to other health records data.	Alvarez-Madrazo et al., Int J Epidem 2016[67]<br>McTaggart et al., Int J Epidem 2018[68]	Part-funded by Farr	13 users<br><br>The PIS has preferred partner status for future pharmacovigilance in collaboration with the European Medicines Agency.
**Smartphones and wearables** Pervasive, continuous and objective remote monitoring through active and passive monitorings	Open source plug'n'play platform for mobile health enabling live monitoring through an extensible platform based on open data standards. Easily adapted for new disorders and devices.<br>Initially developed as part of the IMI2 RADAR-CNS programme.<br>DPUK sensing platform	RADAR-base.org<br>Machin et al., International Journal of Population Data Science 2017[69]	Richard Dobson has been a member of the Farr team since 2015 (jointly with KCL)	Best of show winner at Bio-IT World 2018.<br>Underpinning >£80m of IMI2 investments:<br>RADAR-CNS<br>RADAR-AD<br>BigData@Heart<br>AIMS-2-TRIALS<br>Used in MRC Deep & Frequent Phenotyping study

Table 5. Clinical guideline recommendations informed by Farr research

Farr research	Clinical Guideline Citing Farr Research
**Complying with TB treatment** Farr Institute led the world's first randomised controlled trial of effectiveness of video-observed therapy (VOT) vs directly observed therapy (DOT) for the supervision of tuberculosis treatment.<br>Face to Face Directly Observed Treatment (DOT) which has been the mainstay of tuberculosis treatment for decades. The trial was stopped early due to overwhelming superiority of the intervention arm. Story et al., Lancet 2019 [70]	**World Health Organisation End TB Strategy 2017** Recommendation that Video Observed Therapy (VOT) is a method of supporting patients through tuberculosis treatment using remote observation of treatment with a smart phone app. [70]<br>Commercial platform SureAdhere developed for Tuberculosis, HIV, Hepatitis C and Opioid Substitution Therapy and is beginning to be used in some high incidence countries. http://www.sureadhere.com/
**Zoster vaccination** Farr Institute led one of the very few UK analyses of US (Medicare) data, revealing that herpes zoster vaccination is associated with a reduction in post herpetic neuralgia (PHN) in routine clinical use. As PHN is the major complication of herpes zoster and is associated with highly significant morbidity and adverse impacts on quality of life, guidelines might recommend vaccination and substantial efforts are needed to increase vaccine use in routine care of elderly individuals. Langan et al., PLOS Med 2013 [44]	**UK Joint National Committee on Vaccination and Immunisation, and Centers for Disease Control and other international bodies** have all cited this research informed decisions on introducing the zoster vaccine into routine practice. https://www.cdc.gov/vaccines/acip/recs/grade/herpes-zoster.html [71]<br>http://www.who.int/immunization/sage/meetings/2014/april/2_Background_document_Herpes_Zoster.pdfpage 9 [72]
**High Risk prescribing** Farr Institute research has informed a number high-risk prescribing indicators Marwick et al., BMJ QualSaf. 2014. [73]	**NHS Scotland National Therapeutic Indicators** A number of the researchers' high-risk prescribing indicators have been implemented, http://www.sehd.scot.nhs.uk/publications/DC20151126nti_15-16_Full_Document_FINAL.pdf in NHS prescribing data analysis tools, and e.g. NHS Forth Valley stopped high-risk medicines in 1,200 patients as a result [74]
**IVF Cycle number** Farr investigators analysed 71,551 women using Human Fertilisation and Embryology Authority (HFEA) national database. Most couples in the UK still do not receive 3 IVF cycles; around 83% of women receiving IVF would achieve a live birth by 8th complete cycle, similar to the natural live birth rate in a non-contraception practising population. McLernon et al., Hum Reprod. 2016 [75]	**NHS Scotland** Cited in the Scottish Government National Infertility Group Report where it contributed evidence which led to the recommendation, and subsequent policy change, of increasing the number of NHS funded complete cycles of IVF in Scotland from two to three. Results support the call from NICE to develop consistent IVF policies based on three complete cycles. http://www.gov.scot/Resource/0050/00501403.pdf[76]
**Migrant health** Farr Institute led the development and validated a novel probabilistic data linkage method to create a cohort of over half a million migrants demonstrating that the majority of cases diagnosed in the UK were likely to have resulted from latent infection and made the case for extending screening periods Aldridge et al., Lancet 2016.[77]	**European Centre for Disease Prevention and Control (ECDC)** infectious disease screening guidance (2018 in press)[74]<br>European Centre for Disease Prevention and Control (ECDC) Surveillance report: Tuberculosis surveillance and monitoring in Europe, 2017. Changed screening policy for migrants and evidence of importance of good access to healthcare among migrants.<br>https://ecdc.europa.eu/sites/portal/files/documents/ecdc-tuberculosis-surveillance-monitoring-Europe-2017-WEB.pdf[75]
**Hypertension** Farr Institute led higher resolution estimation of age specific association of blood pressure with a wider range of diseases than hitherto reported. Farr paper cited in 3 separate sections in clinical guideline. Rapsomaniki et al., Lancet 2014 [19]	**American College of Cardiology/American Heart Association Task Force on Clinical Practice Guidelines** 2017 Guideline for the Prevention, Detection, Evaluation, and Management of High Blood Pressure in Adults: Executive Summary https://www.acc.org/~/media/Non-Clinical/Files-PDFs-Excel-MS-Word-etc/Guidelines/2017/Guidelines_Made_Simple_2017_HBP.pdf [78]
**Atrial fibrillation** Farr Institute led the only analysis to report separately in women and men the threshold at which there are net benefits of treating atrial fibrillation with oral anticoagulants. Allan et al., Heart 2016 [79]	The Task Force for the management of atrial fibrillation of the **European Society of Cardiology** (ESC). 2016 ESC Guidelines for the management of atrial fibrillation developed in collaboration with EACTS.
**Stable coronary disease** Farr Institute led a collaboration with Astra Zeneca demonstrating generalisability of the trial population to unselected patients surviving an acute myocardial infarction: event rates of safety and benefits Timmis et al., BMJ 2016. [80]	**NICE.** Ticagrelor for preventing atherothrombotic events after myocardial infarction. Technology appraisal guidance [TA420]. Published 14 December 2016 https://www.nice.org.uk/guidance/TA420/chapter/1-Recommendations[81]
**Genomic medicine** Farr Institute research was cited in the report highlighting importance of longitudinal NHS EHR phenotyping data in delivering vision of genomic medicine. Wu et al., JAMIA 2018 [66]	**Chief Medical Officer Annual Report 'Generation Genome'** the government has establishsed the National Genomics Board chaired by the health minister, to implement recommendations

Table 4. Change in UK training opportunities in data science and health informatics between 2013 and 2018

Level / University	Course title	Year established	Number of students/delegates 2013-2018
**Masters level***			
UCL	Health Informatics	1999	170
UCL	Health Data Science for Research in Biomedicine	2015	74
UCL	Health Data Analytics	2017	36
Swansea University	Health Informatics	2001	176
Swansea University	Health Data Science	2014	88
Manchester University	Health Data Science	2014	60
**Short courses**			
UCL	Farr Institute London 13 short courses (1-3 days)	2014	684
Swansea University	Analysis of linked health data	2008	335
Manchester University	Informatics for Healthcare Systems: Improving Skills for Patient Driven Healthcare	2011	332
**Leadership**			
Edinburgh University /Imperial College†	NHS Digital Academy	2018	105
All 4 Farr Centres: residential meetings in Manchester, Swansea	UK Farr Future Leaders Course	2017	42

*Offered at MSc/Postgraduate Diploma or Postgraduate Certificate levels

†Postgraduate Diploma of Digital Health Leadership

Supplementary Table 1: EHR and administrative data sources reported in the 100 Farr Institute publications

Setting of denominator population / Data Sources	Publication Using the Named Data Sources[a]
**PRIMARY CARE**<br>Clinical research using LInked Bespoke studies and Electronic health Records (CALIBER)[b]	Rapsomaniki et al., Lancet, 2014, PMID: 24881994
UK Clinical Practice Research Datalink (CPRD)	Bhaskaran et al., Lancet, 2014, PMID: 25129328
General practice data (from Public Health England)	Aldridge et al., PLoS One, 2016, PMID: 26933880
General practice data (Scotland)	Mole et al., BMJ Open, 2016, PMID: 27311912
General practice records (in Secure Anonymised Information Linkage databank [SAIL])	Brophy et al., Am J Gastroenterol, 2013, PMID: 23588238
Prescribed medications (in SAIL)	Lyons et al., J Epid Comm Health, 2016, PMID: 27217535
Prescribing Information System (PIS, for Scotland)	Black et al., PLoS Med, 2016, PMID: 26978456
Primary Care Clinical Information Unit Research database (PCCIUR)	Busby et al., Int J Cancer, 2017, PMID: 28120338
Wales Demographic Service Dataset (WDS)	Paranjothy et al., Lancet, 2018, PMID: 24249824
Wales Electronic Cohort for Children (WECC, in SAIL)	Morgan et al., PLoS One, 2013, PMID: 24236160
WECC(4)[c]	Hutchings et al., Pediatrics, 2016, PMID: 23940601
WECC(6)[d]	Paranjothy et al., Pediatrics, 2013, PMID: 24249824
WECC(8)[e]	Hutchings et al., PloS One, 2013, PMID: 23940601
**HOSPITAL (DETAILED DATA)**	
Aberdeen Maternity and Neonatal Databank (AMND)	Clemens et al., Environ Int, 2017, PMID: 28753483
Antenatal care (Wales)	Hurt et al., Prenat Diagn, 2015, PMID: 26475362
Clinical Record Interactive Search (CRIS)	Iqbal et al., PLoS One, 2015, PMID: 26273830
Genetic service laboratories (Scotland)	Jacobs et al., PLoS One, 2016 , PMID: : 27907018
Intensive Care National Audit & Research Centre data	Mukherjee et al., BMC Med, 2016, PMID: 27568881
Medicare (United States)	Langan et al., PLoS Med, 2013, PMID: 23585738
Paediatric Intensive Care Audit Network (PICANet)	Gilbert et al., Lancet, 2016, PMID: 26946925
Single Clinical Biochemistry Service (from NHS Grampian)	Marks et al., Nephrol Dial Transplant, 2015, PMID: 25943597
**HOSPITAL (LIMITED CODED DATA)**	
A&E Datamart	Mukherjee et al., BMC Med, 2016, PMID: 27568881
A&E2 database	Mole et al., BMJ Open, 2016, PMID: 27311912
Adult ICU data (Cambridge; Guy's; King's and St Thomas'; Imperial; Oxford and UCL)	Harris et al., Int J Med Inform, 2018, PMID: 29500026
Child's hospital administrative records (CATheter Infections in Children [CATCH] trial)	Gilbert et al., Lancet, 2016, PMID: 26946925
Emergency Department Dataset (in SAIL)	Mukherjee et al., BMC Med, 2016, PMID: 27568881
Hospital data (Diana Princess of Wales Hospital; Scunthorpe General Hospital; Scarborough Hospital; and York Hospital)	Mohammed et al., J Health Serv Res Pol, 2017, PMID: 29944016
Hospital data (Denmark)	Schmidt et al., Am J Epidemiol, 2018, PMID: 29053820
Hospital Episode Statistics (HES)	Herbert et al., PLoS Med, 2015, PMID: 26714280
Maternity record data (Manchester)	Kelly et al., Autism, 2017, PMID: 29113453
Mental health data (in SAIL)	White et al., Am J Epidemiol, 2017, PMID: 28486637
Out-patient datasets (in SAIL)	Brophy et al., Am J Gastroenterol, 2013, PMID: 23588238
Patient Episode Database for Wales (PEDW, in SAIL)	Morgan et al., BMJ open, 2014, PMID: 24236160
Scottish Morbidity Record 00 (Outpatient Attendance)	Mole et al., BMJ Open, 2016, PMID: 27311912
Scottish Morbidity Record 01 (General/Acute Inpatient and Day Case data set)	Black et al., PLoS Med, 2016, PMID: 26978456
Scottish Morbidity Record 02 (Maternity Inpatient and Day Case)	Black et al., PLoS Med, 2016, PMID: 26978456
Scottish Morbidity Record 04 (Mental Health Inpatient and Day Case)	Mole et al., BMJ Open, 2016, PMID: 27311912
Ultrasound scan (a Welsh NHS hospital)	Hurt et al., Prenat Diagn, 2015, PMID: 26475362
**registries of diseases and procedures**	
British Cardiovascular Intervention Society database (BCIS)	Hulme et al., CircCardiovascInterv, 2017, PMID: 28196898
Danish National Diabetes Register (NDR)	Schmidt et al., Am J Epidemiol, 2018, PMID: 29053820
Danish National Patient Registry	Schmidt et al., Am J Epidemiol, 2018, PMID: 29053820
Danish National Prescription Registry	Schmidt et al., Am J Epidemiol, 2018, PMID: 29053820
Myocardial Ischaemia National Audit Project (MINAP)	Chung et al., Lancet, 2014, PMID: 24461715
National Adult Cardiac Surgery Audit (NACSA)[f]	Hickey et al., JAMA Intern Med, 2017, PMID: 27820610
National Registry of Childhood Tumours (NRCT)	Williams et al., N Engl J Med, 2013, PMID: 24195549
Psychiatric Central Research Register (PCRR)	Schmidt et al., Am J Epidemiol, 2018, PMID: 29053820
Renal management system (from NHS Grampian)	Marks et al., NephrolDialTransplant, 2015, PMID: 25943597
Respiratory Datamart data	Aldridge et al., PLoS One, 2016, PMID: 26933880
Scottish Care Information Diabetes Collaboration (SCI-DC)	Black et al., PLoS Med, 2016, PMID: 26978456
Scottish Intensive Care Society Audit Group (SICSAG)	Mole et al., BMJ Open, 2016, PMID: 27311912
Scottish Morbidity Record 06 (Scottish Cancer Registry)	Black et al., PLoS Med, 2016, PMID: 26978456
Scottish Renal Registry (SRR)	Marks et al., Nephrol Dial Transplant, 2015, PMID: 25943597
Scottish Stroke Care Audit (SSCA)	Turner et al., J Neurol Neurosur Psyc, 2015, PMID: : 24966391

Setting of denominator population / Data Sources	Publication Using the Named Data Sources[a]
Sentinel Stroke National Audit Programme (SSNAP)	Bray et al., Lancet, 2016, PMID: 27178477
Swedish Web-System for Enhancement and Development of Evidence-Based Care in Heart Disease Evaluated According to Recommended Therapies / Register of Information and Knowledge about Swedish Heart Intensive care Admissions (SWEDEHEART/RIKSHIA)	Chung et al., Lancet, 2014, PMID: 24461715
United Kingdom Human Fertilisation and Embryology Authority (HFEA)	Williams et al., N Engl J Med, 2013, PMID: 24195549
National Data Bank for Rheumatic Diseases (United States)	Movahedi et al., Arthritis Rheumatol, 2016, PMID: 26663814
**DEATH DATA**	
Death certification records (from the CATCH trial)	Gilbert et al., Lancet, 2016, PMID: 26946925
Mortality data (in SAIL)	Rahman et al., Br J Psychiatry, 2018, PMID: 29506597
National Death Registry (Sweden)	Chung et al., Lancet, 2014, PMID: 24461715
Office for National Statistics (ONS) data	Hall et al., JAMA, 2016, PMID: 27574717
Scottish Health and Ethnicity Linkage Study (SHELS)[g]	Gruer et al., J Epidemiol Community Health, 2016, PMID: 27473157
Scottish Morbidity Record 99 (Death Registrations)	Mole et al., BMJ Open, 2016, PMID: 27311912
**OTHER HEALTH**	
All Wales Injury Surveillance System (AWISS)[h]	Lyons et al., Inj Prev, 2015, PMID: 26658339
Caerphilly County Borough Council (Wales)	White et al., Am J Epidemiol, 2017, PMID: 28486637
Electronic Communication of Surveillance in Scotland (ECOSS)	Kavanagh et al., J Antimicrob Chemo, 2017, PMID: 27999064
Enhanced Tuberculosis Surveillance system (ETS)	Aldridge et al., Lancet, 2016, PMID: 27742165
Health and Occupation Research Network	Mukherjee et al., BMC Med, 2016, PMID: 27568881
Health Intelligence (from NHS Grampian)	Marks et al., NephrolDialTransplant, 2015
National Community Child Health Database (NCCHD)	Morgan et al., PLoS One, 2013
NHS-24	Mukherjee et al., BMC Med, 2016, PMID: 27568881
Practice Team Information (PTI)	Mukherjee et al., BMC Med, 2016, PMID: 27568881
Salford Integrated Record (SIR)[i]	Fraccaro et al., BMC Med, 2016, PMID: 27401013
Scottish Care Information (SCI) Stores	Mole et al., BMJ Open, 2016, PMID: 27311912
Support Needs System (SNS)	Black et al., PLoS Med, 2016, PMID: 26978456
**SOCIO-ECONOMIC AND ENVIRONMENT**	
Demographic data (from NHS Scotland)	Bergman et al., BMC Cancer, 2016, PMID: 27178424
General Register Office (GRO, for Scotland) data	Turner et al., J Neurol Neurosurg Psychiatry, 2015, PMID: 24966391
National Records of Scotland (NRS) data	Black et al., PLoS Med, 2016, PMID: 26978456
Northern Ireland Statistics data	Mukherjee et al., BMC Med, 2016, PMID: 27568881
Annual pupil census (Scotland)	Mackay et al., Am J Epidemiol, 2016, PMID: 27651381
Child Health Systems Programme (CHSP) School	Black et al., PLoS Med, 2016, PMID: 26978456
Danish Population Education Register (PER)	Schmidt et al., Am J Epidemiol, 2018, PMID: 29053820
Education records (in SAIL)	Rahman et al., Br J Psychiatry, 2018
School attainment record (Scotland)	Tweed et al., Arch Dis Child Fetal Ne Ed, 2015
Air pollution data	Lyons et al., J Epid Comm Health, 2016
Local weather data	Druce et al., JMIR Mhealth Uhealth, 2017, PMID: 29092810
United Kingdom Atomic Energy Authority (AEA)	Dibben et al., Environ Res, 2015, PMID: 26005952
United Kingdom Department for the Environment, Food and Rural Affairs (DEFRA) data	Clemens et al., Environ Int, 2017, PMID: 28753483
Department of Work and Pensions (DWP) data	Mukherjee et al., BMC Med, 2016, PMID: 27568881
Index of Multiple Deprivation (IMD)	Conrad et al., Lancet, 2017
Weekly Returns Service (WRS)	Mukherjee et al., BMC Med, 2016, PMID: 27568881
Welsh Costing Return (WCR)	Morgan et al., BMJ open, 2014
Administrative data (crime rate from the Scottish Longitudinal Study [SLS] study)	Dibben et al., Environ Res, 2015, PMID: 26005952
Global Positioning System (for smartphone)	Druce et al., JMIR Mhealth Uhealth, 2017, PMID: 29092810
2001 census data	Bhopal et al., Eur J Public Health, 2015, PMID: 25888579
Census records (from the SLS study)	Clemens et al., Eur J Public Health, 2017, PMID: 28753483
Department for Social Development data (Northern Ireland)	Mukherjee et al., BMC Med, 2016, PMID: 27568881
Department of Finance and Personnel data (Northern Ireland)	Mukherjee et al., BMC Med, 2016, PMID: 27568881
Department of Health Social Service and Public Safety data (Northern Ireland)	Mukherjee et al., BMC Med, 2016, PMID: 27568881
International Organization for Migration database (IOM) data	Aldridge et al., Lancet, 2016, PMID: 27742165
Swansea Social Services data	Mukherjee et al., BMC Med, 2016, PMID: 27568881
Tweets	Patel et al., npj Digital Medicine, 2018

Notes:a. Multiple datasets are reported together in b. CALIBER [4]: CPRD, HES, MINAP, ONS mortality and index of multiple deprivation c. WECC [4]: the Welsh Demographic Service (WDS), the National Community and Child Health Database (NCCHD), the Office for National Statistics (ONS), the Patient Episode Database for Wales (PEDW); d. WECC [6]: Public Health Birth files from the Office for National Statistics, National Community Child Health Database, Public Health Mortality files from the Office for National Statistics, Patient Episode Dataset for Wales, All Wales Perinatal Survey, Congenital Anomaly Register and Information Service; e. WECC [8]: General Practice data, the Welsh Demographic Service (WDS), the National Community and Child Health Database (NCCHD), the Office for National Statistics (ONS), the Patient Episode Database for Wales (PEDW), the Congenital Anomaly Register and Information Services (CARIS), the National Pupil Database (NPD), the Pupil Level Annual School Census (PLASC); f. NACSA [3]: Life status, surgical

reoperation, and Transcatheter Aortic Valve Implantation (TAVI); g. SHELS [2]: death records (Scotland), 2001 census records (Scotland); h. AWISS [3]: emergency department attendances, general practice events, and hospital admissions**;** SIR [2]: data from 53 primary care providers and 1 secondary care provider.

Supplementary Figure 1: Farr Institute allocation of the £18m revenue, £20m capital and £1m network

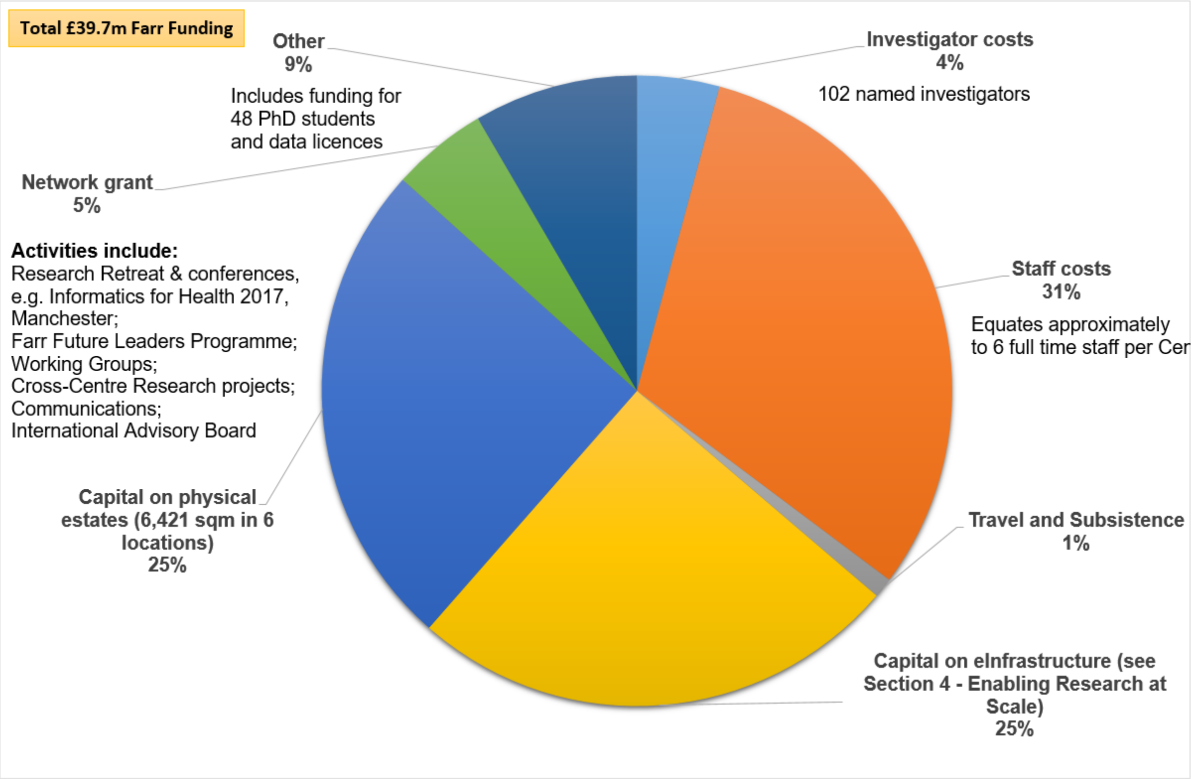

Supplementary Figure 2: Timeline: evolution from 4 separate centres to Farr as a single national institute

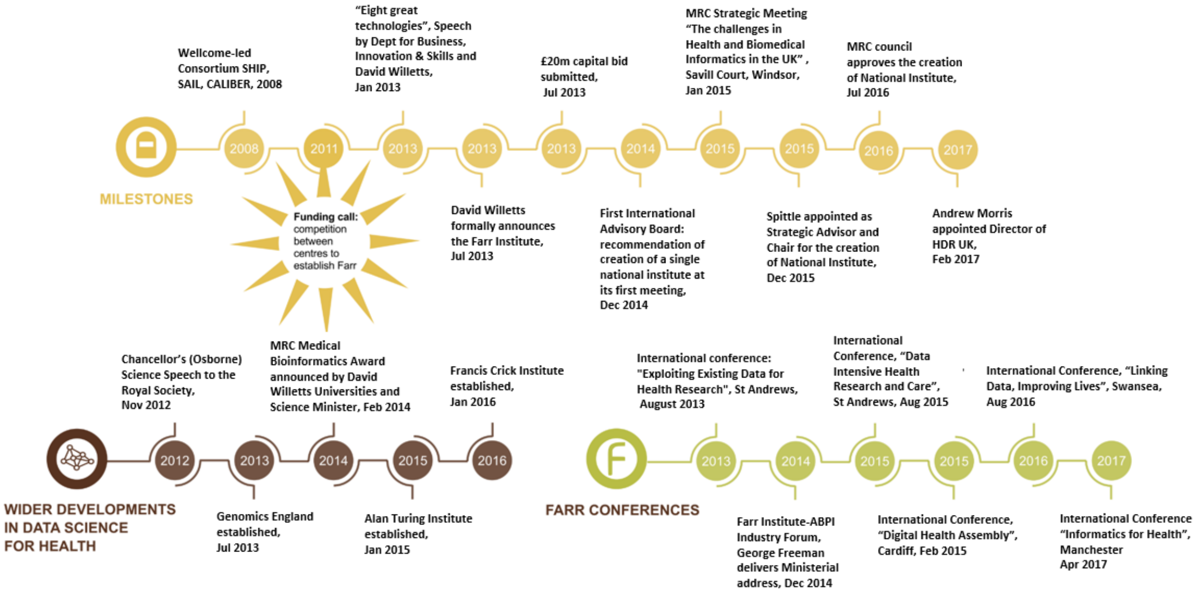

Supplementary Figure 3: Timeline: information governance scandals and policy context shaping evolution of Farr

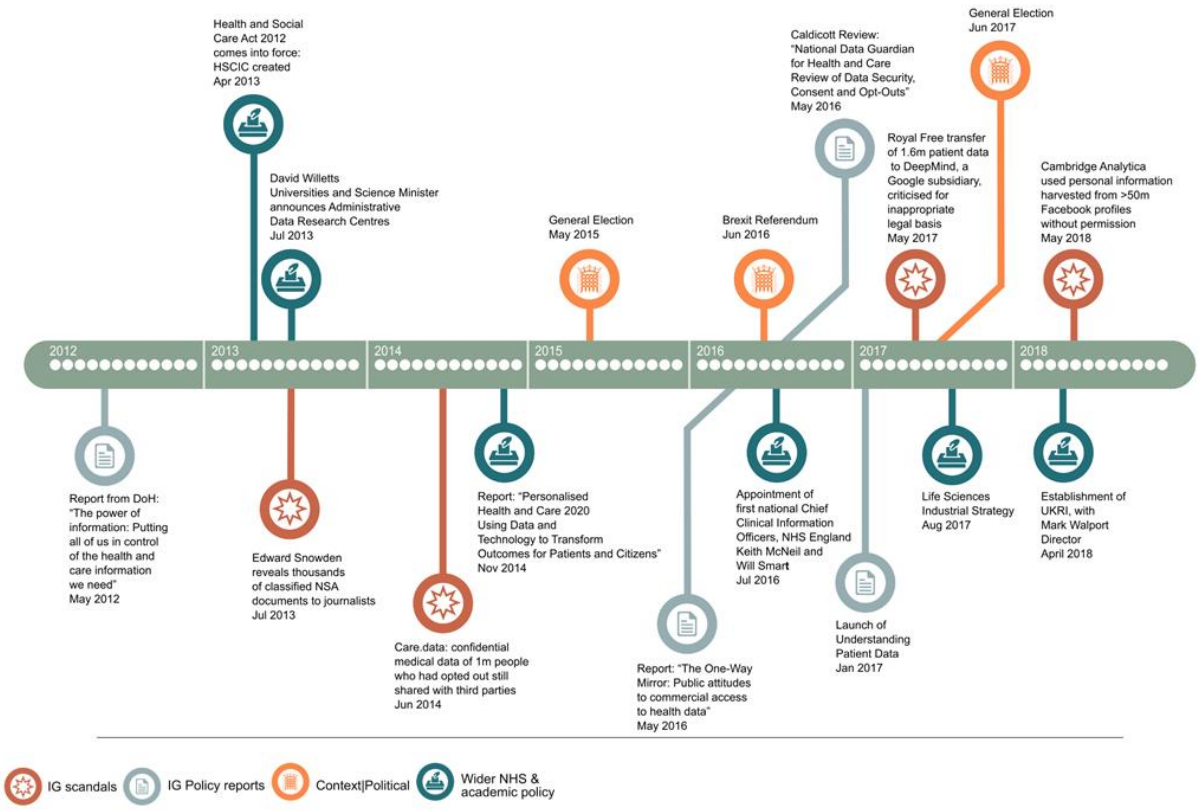

Supplementary Figure 4: Attribution of research publications to the Farr Institute

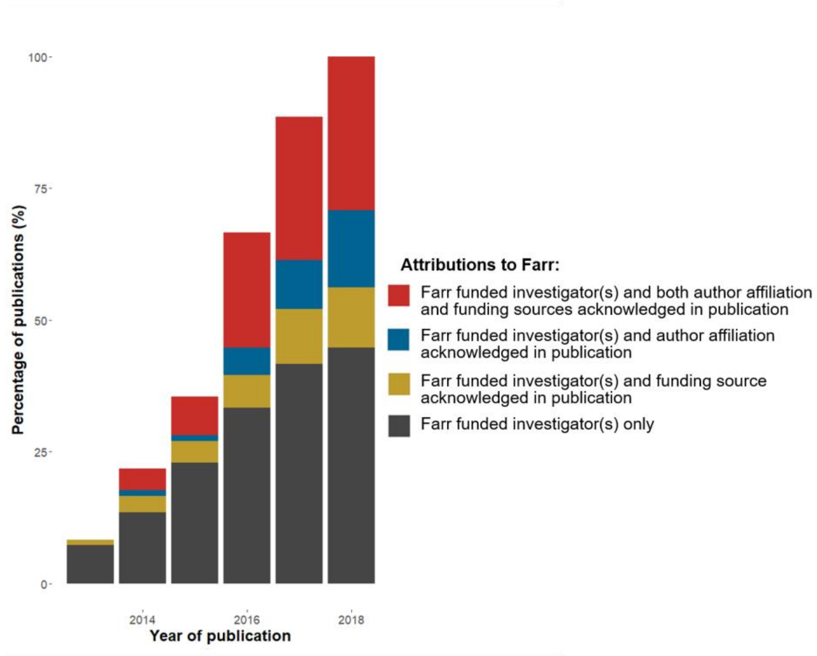

Supplementary Figure 5: Change in inter-disciplinarity based on departmental affiliations: first 30 months and final 30 months

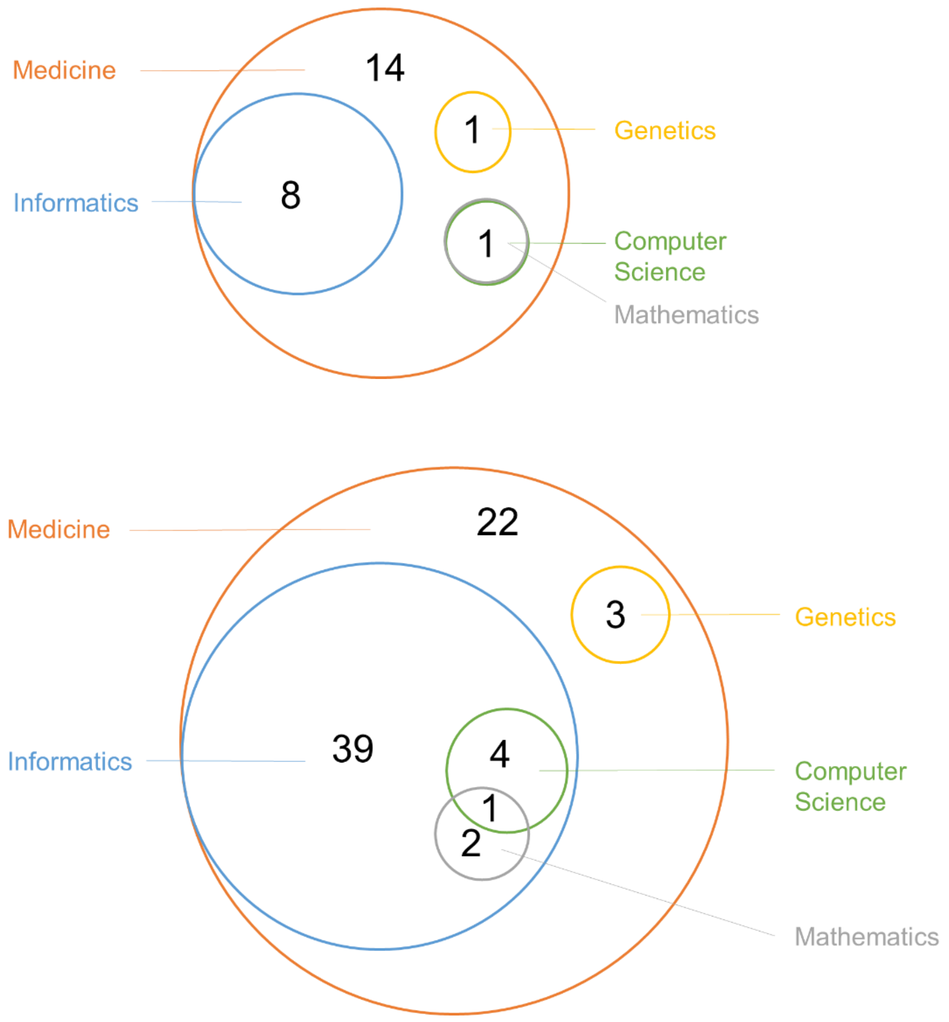

Supplementary Figure 6: Number of institutions collaborating per publication over time*

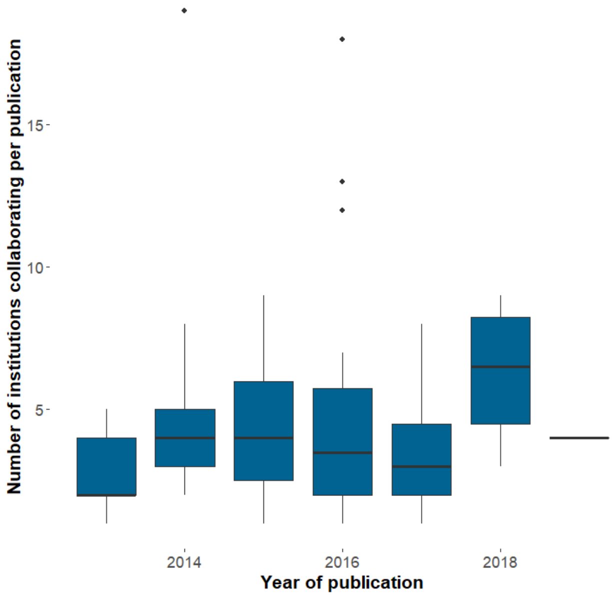

* **Based on those Farr 100 publications with a record in Scopus (n=87) allowing unique identification of institutions.**

Supplementary Box: Original funding call from the UK Medical Research Council (2011) which led to the establishment of the Farr Institute

E-Health Informatics Research Centres (E-HIRCs) Call

It is vital that the UK research community is in a strong position to maximise the health research potential offered by linking electronic health records with other forms of routinely collected data and research datasets. The major funders of health research are working in partnership to ensure the UK builds critical mass and expertise in health informatics research. This call to establish centres of excellence in research using e-health records is part of a shared vision to promote linkage of electronic data for research.

Aim of the Call

The Medical Research Council, in partnership with Arthritis Research UK, the British Heart Foundation, Cancer Research UK, the Economic and Social Research Council, the Engineering and Physical Sciences Research Council, the National Institute of Health Research, the National Institute for Social Care and Health Research (Welsh Assembly Government), the Chief Scientist Office (Scottish Government Health Directorates) and the Wellcome Trust invite proposals for Centres of Excellence in research linking electronic health data. The Centres will pursue cutting-edge research, deliver innovative linkage and analysis of health-related data sets, build research capacity and expertise and act as an interface with industry, practice and policy with the aim of building and sustaining a vibrant health informatics research capability in the UK.

Background

The UK has an international reputation for undertaking leading edge large scale health data analysis and population based research. New infrastructure initiatives across the UK1, designed to make electronic health records available for research purposes in a secure and ethical environment, will provide unprecedented opportunities for clinical, health services, social and public health research. In recognition of the vast research potential offered by e-health records, the major funders of e-health records research2 came together under the Office for Strategic Coordination of Health Research (OSCHR) to develop an e-health Research Framework3. To understand how well the UK was placed to capitalise on these opportunities, the MRC led a mapping exercise4 on behalf of the UK research funders and the Association of British Pharmaceutical Industries (ABPI) to review the existing UK capability and examine the requirements to support a

[1] The NIHR Research Capability Programme in England, the Scottish Health Informatics Programme (SHIP) and the Welsh Secure Anonymised Information Linkage system (SAIL)
[2] Cancer Research UK (CRUK), Chief Scientist Office, Scottish Government Health Directorates (CSO), Engineering and Physical Sciences Research Council (EPSRC), Economic and Social Research Council (ESRC), Medical Research Council (MRC), National Institute for Health Research (NIHR), National Institute for Social Care and Health Research (NISCHR), The Wellcome Trust

[3] http://www.mrc.ac.uk/Utilities/Documentrecord/index.htm?d=MRC006669
[4] Http://www.mrc.ac.uk/e-health

sustainable research base in the future5. The report highlighted the need to build capability and capacity in health informatics research and for further methodological development in complex data linkage.

The wealth of scientific possibilities and huge benefits for biomedical and social sciences offered by data linkage were further highlighted at a recent Frontiers meeting hosted by the funders6. In response to these inputs the funders have agreed to jointly fund a number of centres of excellence in health informatics research across the UK with the aim of conducting leading research and building capacity in the area.

Expectations of Centres

The objectives of the Centres will be to:

- provide a focus for high-quality, cutting-edge research using electronic health records;
- undertake and promote innovative linkage and analysis of large health related data sets including social and economic data;
- build capacity in electronic health informatics research.

Centres will either be based around an existing research group(s) or research unit(s) in a single institution or bring together expertise across several institutes in a wider consortium. By building on existing research strengths, Centres will be well placed to provide UK leadership in the area. Centres will create UK capability in electronic health informatics research by providing advice and expertise to support the wider research community, encouraging collaboration across the biomedical, social and computer sciences and offering training and career development opportunities.

Centres will focus on research to improve the health and wellbeing of the population or address major challenges arising from conditions of significant health burden such as cancer, cardiovascular disease, musculoskeletal conditions, neurodegeneration and mental health.

*Each Centre must meet the following **ESSENTIAL** criteria:*

Scientific Excellence

- Existing strengths in health-related informatics research in a recognised research environment; *and*
- Innovative programmes of research which involve linkage between electronic health records or linkage of electronic health records to other datasets including research data, geo-spatial and socio-economic records. Use of existing or emerging e-health records infrastructures must be core to research activities; *and*
- Develop and apply new methods for data manipulation, linkage or analysis in key areas of statistics, computer science or informatics.

Training and career development

[5] Http://www.mrc.ac.uk/e-health

[6] Wellcome, ESRC and MRC

- Offer training for PhD students, post-doctoral fellows and visiting workers where skills can be learnt on the job; *and*
- Provide career development opportunities for enabling roles such as data managers, software engineers, informaticians and data analysts; *and*
- Create an environment to develop and sustain capacity in research using e-health records.

Management structures and Governance

- Each Centre will have a Director based within the lead host institution who will take responsibility for the delivery of the objectives of the Centre.
- The Director will be a recognised leader in health informatics research.
- Centres must have a clear governance structure with well articulated decision making processes.
- Senior management roles and responsibilities must be clear.

Networking activities

- Provide scientific leadership, support and advice to the wider research community on the design, linkage, conduct and analysis of e-health related research.
- Contribute to networking activities with the other e-health research Centres of excellence designed to strengthen the national capability in e-health records research.

Public engagement

Centres must engage patients and the public individually or collectively in research and undertake a programme of activities to promote public and professional understanding of the health and societal benefits of e-health records research.

All Centres will be required to demonstrate they meet the essential criteria but it is recognised that individual Centres may not have equal strengths in each of the criteria.

Additional Strengths

In addition to meeting the essential criteria the funders welcome applications which address any of the following:

- Establish a modular training programme in e-health records research that would assist individuals from different backgrounds to 'top up' their skills.
- Issues relating to ethics, security, anonymisation and governance surrounding data linkage.
- Develop innovative linkage across different types of data such as omics and imaging data, and other non-health datasets including demographic, geo-spacial and socio-economic data.
- Translate research outputs by developing effective links with the NHS, policy makers, the public and industry including secondments and temporary placements and 'case type awards'.

- Create and provide access to datasets for secondary analysis by the research community where legally and ethically possible.

Sustainability

In line with the funders' desire to build long term capability in e-health informatics research in the UK, Centres will be required to demonstrate commitment from the host HEI(s) to maintain the capacity and expertise developed by the Centre, both in academic and enabling 'non-academic' positions, beyond the term of the five year Centre award. A supporting statement including a sustainability plan from the host institution(s) will be required (from the Vice Chancellor or equivalent).

A UK network of E-HIRCs

Individual Centres will be networked together to further strengthen the national capability for using electronic health data in research. The UK network of E-HIRCs will facilitate information exchange about research opportunities and best practice including consideration of ethics and governance issues. The Network will provide a focus for networking with the wider research community and encourage cross-Centre collaborative working. Each Centre will be expected to collaborate on coordinating workshops, seminars, web-based resources, etc to ensure the UK at the leading edge of the field.

Collectively the UK network of E-HIRCs will accelerate the translational process through the development of innovative and transformative approaches for the linkage of data, as well as the design, conduct and analysis of research using electronic health records.

Once the Centres are awarded, a nominated 'lead' centre will be allocated a budget in the vicinity of £1m to coordinate activities. Applicants can bid in their proposals to undertake this role.

Funding available

Funds of ~£15m are available to support a number of Centres across the UK for up to five-years and awards will be made on the basis of full economic costs (fEC) at approximately 67% to reflect collaborative funding from research councils, charities and government departments. As these Centres will represent leading foci of expertise it is expected that requests for support will be for a minimum of £3m over the funding period, although less may be awarded as determined by rigorous peer-review. In all areas, the resources requested must be fully justified as part of a coherent scientific programme; the quality of the science proposed will be key.

Following completion of the five year period, a funding extension that tapers over the next 5 years may be considered if good progress has been made. However a long term sustainability plan with demonstrable commitment by the host HEI to ongoing support of the Centre at the end of the initial 5 year funding term must be part of the Centre application.

Awards for Centres will be managed by the MRC on behalf of the partners and subject to MRC terms and conditions.

Support available

- The Director's salary costs for the portion of their time dedicated to scientific and management leadership of the Centre;
- Funds for research, in particular to support new collaborations across and outside the Centre, although it is anticipated that most of the research in any Centre will be supported by successful applications to response mode competitions.
- Funds to enable the Centre to function as a cohesive regional or UK focus for e-health research (e.g. funds for pilot studies, support posts, networking costs, common resources/datasets and equipment etc);
- Start-up costs for new positions to develop capacity (this can include the initial costs of new appointments from overseas) or key positions central to the Centre's success;
- Funds to provide a support/advisory service to collaborate with researchers working outside the Centre wishing to use electronic health care records for research;
- Funds for training and capacity building in methodologies underpinning linking and the use of electronic health records. Centres are eligible for MSc and PhD studentships in areas were unmet need is clearly articulated.
- Dedicated Centre management and administrative staff
- Other resources essential to establish and maintain the Centre;
- Public and user engagement, coordination and collaboration costs to bring the research closer to the patient or to application.
- Translational activities for building collaborations with industry, NHS, etc
- Resources to undertake activities as part of the wider UK network. If wishing to take the lead, a request for a coordinator can be made.

Support not available

Funds cannot be requested for capital and refurbishment costs or infrastructure and core administrative services (secretarial, finance, personnel, computing support, estates and building maintenance) covered by the indirect cost component of FEC.

New activities and facilities will only be funded where they are clearly not attributable to existing grants. The funding partnership will not provide support for any shared infrastructure.

Eligibility

For administrative purposes, the E-HIRC application will be led by a single UK academic institution or academic analogue approved by the Research Councils7.

[7] http://www/rcuk.ac.uk/research/Pages/Eligibilityforrcs.aspx

Only one proposal is permitted per applying host institution. Bids involving multiple sites are welcomed but will be required to demonstrate the added value of the consortium arrangement and how effective working across sites will be achieved. Applications throughout the UK are eligible.

The Director must be the principal investigator on the proposal and be given appropriate status within the lead host institution. The Director should be in a position to influence research programmes associated with the Centre, and must have full control over use of Centre Grant funds.

Co-applicants are expected to be the leading scientists involved in delivering the Centre's aims and objectives.

The normal MRC eligibility rules apply; please see the applicants' handbook and the Research Council UK website.

Partnerships with industry are welcome. Applicants considering establishing a collaboration with an industrial partner(s), are advised to refer to the guidance on MRC Industry Collaboration Awards (MICAs),

Application and assessment process

Applicants will submit outline proposals on the RCUK Je-s application system. Specific instructions for completing the case for support are detailed in the Guidance Notes for e-Health Informatics Research Centres [insert link]. Outline proposals will be reviewed by an expert Scientific Panel and successful bids will be invited to submit full proposals. The panel will encourage collaboration across bids where appropriate. Full proposals will be externally peer reviewed before being considered by the Scientific Panel. Applicants will be required to present their application to the Panel before a final recommendation for funding is made. Final funding decisions will be made by the contributing funding partners.

The Host Institution(s) will be expected to identify their own targets and milestones for the Centre and report to funders on an annual basis. Centres will also be required to annually submit reports to MRC's Electronic Valuation System (e-Val).

ALL applicants are required to discuss their proposals with the Programme Manager before submission. A brief abstract should be e-mailed in advance of discussions.

Programme Manager:

Dr Mark Pitman

E-Mail: mark.pitman@headoffice.mrc.ac.uk

Tel: 0207 395 2215

Timetable

Task	Deadline
Call Launch	6th September 2011
Deadline of Outline Applications	1st November 2011
Panel Meeting (Outlines)	9th December 2011
Deadline of Full Applications	27th February 2012
Scientific Panel meeting with interviews	June 2012

A national initiative in data science for health: an evaluation of the UK Farr Institute

Acknowledgements
The funders of the Farr Institute were: Medical Research Council (co-ordinating), Arthritis Research UK, British Heart Foundation, Cancer Research UK, Chief Scientist Office, Economic and Social Research Council, Engineering and Physical Sciences Research Council, National Institute for Health Research, National Institute for Social Care and Health Research, and Wellcome Trust.

Farr Institute
The work of the Farr Institute was carried out by a network of about 430 investigators, students and professional support staff: the majority of whom received either no, or limited direct funding support from the Farr Institute award for their salaries, as follows:

International Advisory Board

Nancy Pedersen (Chair), Professor in Genetic Epidemiology, Department of Medical Epidemiology and Biostatistics, Karolinska Institute, Sweden, Director of LifeGene project

Dan Roden, Assistant Vice Chancellor for Personalized Medicine Vanderbilt University Medical Center

Phil Burstein, Vice President, Health Care Data Optimisation and Data Stewardship Operation, Drug Development Sciences, GlaxoSmithKline

Georges De Moor, Immediate Past President and Member of the Board of European Institute for Health Records and Head of the Department of Medical Informatics and Statistics at the State University of Ghent, Belgium

Ian Crichton, Managing Director UK Health Business, Serco

Simon Denegri, Chair of INVOLVE and NIHR's National Director for Public Participation and Engagement in Research

Isaac Kohane, Professor of Pediatrics and Health Sciences and Technology, Harvard Medical School, Chair, Informatics Program, Boston Children's Hospital, Children's Hospital Informatics Program (CHIP)

William Lowrance, Consultant in Health Research Ethics & Policy, La Grande Motte France, and author of Privacy and Confidentiality in Health Research

Michael Parker, Professor of Bioethics and Director of the Ethox Centre, University of Oxford

John Speakman, Senior Director, Research Information Technology, NYU Langone Medical Center

Graham Spittle, Chief Technology Officer Europe & Vice President, Software Group, IBM

Matthew Swindells, NHS England National Director for Commissioning Operations and Information

Bart Vannieuwenhuyse, Senior Director Health Information Sciences, Janssen and European Medical Information Framework (EMIF) Coordinator

Directors (funded 0.4 WTE)

Harry Hemingway, Professor of Clinical Epidemiology, Research Director, HDR UK London; Director, UCL Institute of Health Informatics, 222 Euston Road, London NW1 2DA

Ronan Lyons, Clinical Professor of Public Health Research, Director HDRUK Wales/Northern Ireland; Swansea University Medical School, Fourth Floor, Data Science Building, Singleton Campus, Swansea, SA2 8PP

Iain Buchan, Professor of Health Informatics, North England, University of Liverpool, Liverpool L69 3BX
John Ainsworth, Professor of Health Informatics, Division of Informatics, Imaging & Data Sciences, The University of Manchester, Oxford Rd, Manchester M13 9PL
Jill Pell, Henry Mechan Professor of Public Health, Director, Institute of Health and Wellbeing, University of Glasgow, 1 Lilybank Gardens, Glasgow G12 8RZ
Andrew Morris, Professor of Medicine, University of Edinburgh, Director, HDR UK

Deputy Directors (funded 0.2 WTE)

Corri Black, Deputy Director, Farr Institute of Health Informatics, Scotland; Clinical Professor, Aberdeen Centre for Health Data Science, 1:042 Polwarth Building, School of Medicine, Medical Science and Nutrition, University of Aberdeen, Foresterhill, Aberdeen
David Ford, Deputy Centre Director, Farr Institute of Health Informatics, Wales; Professor of Health Informatics, Swansea University Medical School, Fourth Floor, Data Science Building, Singleton Campus, Swansea
Tjeerd van Staa, Deputy Director, Farr Institute of Health Informatics, North England; Professor in eHealth Research, Division of Informatics, Imaging & Data Sciences, The University of Manchester, Oxford Rd, Manchester
Liam Smeeth,Deputy Director, Farr Institute of Health Informatics, London; Professor of Clinical Epidemiology, Department of Non-communicable Disease Epidemiology, London School of Hygiene & Tropical Medicine, Keppel Street, London

Research investigators (not listed above), nearly all of whom held substantive academic appointments in universities (funded 0.02 WTE)

Michael R Barnes, Reader in Bioinformatics, Queen Mary Universityof London
Helen Bedford, Professor of Children's Health, University College London
Marion Bennie, Professor of Pharmacy Practice in SIPBS, University of Strathclyde
Ann Blandford, Professor of Human Computer Interaction, University College London
Andy Briggs, Professor. Chair in Health Economics (Health Economics and Health Technology Assessment), University of Glasgow
Peter Brocklehurst, Professor of Women's Health, University College London (Currently Birmingham)
Sinead Brophy, Professor of Data Science, Swansea University
Gavin Brown, Professor of Machine Learning, University of Manchester
Paul Burton, Professor of Infrastructural Epidemiology, University of Leicester
Paul Burton, Professor of Infrastructural Epidemiology, University of Bristol
Christopher Butler, Professor of Primary Care Cardiff, University
Simon Capewell, Professor of Clinical Epidemiology, University of Liverpool
James Carpenter, Professor of Medical Statistics London, School of Hygiene and Tropical Medicine
John Carroll, Professor of Computational Linguistics (Informatics), University of Sussex
Jackie A. Cassell, Professor of Primary Care Epidemiology, Brighton and Sussex Medical School

Fortunato Castillo, Chief Information Scientist, MRC Centre of Epidemiology for Child Health, Chief Information Officer, Life Study, UCL Institute of Child Health

Mike Catchpole, Director of the Centre for Infectious Disease Surveillance and Control Public Health England

Mark Caulfield, Professor of Cardiovascular Genetics, Queen Mary University of London

Helen Colhoun, Professor. Chair in Medical Informatics and Life Course Epidemiology, University of Dundee

Peter Coveney, Professor in Computer Science, University College London

Sarah Cunningham-Burley, Professor of Medical and Family Sociology, University of Edinburgh

Adnan Custovic, Clinical, Professor of Paediatric Allergy, University of Manchester

John Deanfield, Professor of Cardiology, University College London

Spiros Denaxas, Senior Lecturer, University College London

Michael Dennis, Professor of Biostatistics and Epidemiology, Swansea University

Carol Dezateux, Professor of Clinical Epidemiology and Health Data Science, University College London

Chris Dibben, Professor of Health Geography, University of Edinburgh

Peter Diggle, Professor of Epidemiology and Statistics, University of Liverpool/Lancaster

Will Dixon, Professor of Digital Epidemiology, University of Manchester

Graham Dunn, Professor of Biomedical Statistics, University of Manchester

Khaled El Emam Chair in Electronic Health Information, University of Ottawa

David Fone, Professor Cardiff, University

David Ford, Deputy Centre Director, Swansea University

Ian Ford, Professor of Biostatistics, University of Glasgow

John Frank, Chair, Public Health Research and Policy, University of Edinburgh

Nick Freemantle, Professor of Clinical Epidemiology & Biostatistics, University College London

Belinda Gabbe, Head of the Pre-Hospital, Emergency and Trauma Research Unit in the Department of Epidemiology and Preventive Medicine, Monash University

John Gallacher, Professor of Cognitive Health, University of Cardiff

Martin Gibson, Professor/Consultant in Diabetes/Endocrinology, University of Manchester

Ruth Gilbert, Professor of Clinical Epidemiology, University College London

Mika Gissler, Professor THL (Finnish National Institute for Health and Welfare)

Carol Goble, Professor of Computer Science, University of Manchester

Andy Goldberg Clinical Senior Lecturer, University College London

Mike Gravenor, Professor of Biostatistics and Epidemiology, Swansea University

David Gunnell, Professor of Epidemiology, University of Bristol

Phil Hannaford, Professor of Primary Care, University of Aberdeen

Andrew Hayward, Professor of Infectious Disease Epidemiology and Inclusion Health, University College London

Matthew Hickman, Professor in Public Health and Epidemiology, University of Bristol

Aroon Hingorani, Professor Genetic Epidemiology, University College London

Soren Holm, Professor of Bioethics, University of Manchester

Cashel Holman, Emeritus, Professor/Senior Honorary Research Fellow, University of Western Australia

Gareth John, Senior Statistician, NHS Wales Informatics Service

Ann John, Associate Professor of Public Mental Health, Swansea University

Kerina Jones, Associate Professor of Health Informatics, Swansea University

Dipak Kalra, Clinical Professor of Health Informatics, University College London

Graeme Laurie, Professor of Medical Jurisprudence, University of Edinburgh

Shon Lewis, Professor of Adult Psychiatry, University of Manchester

Keith Lloyd, Professor of Clinical Psychiatry, Swansea University

Sarah Lowe, Senior Research Officer Welsh Government

Colin McCowan, Professor of Health Informatics, University of Glasgow

John Macleod, Professor of Clinical Epidemiology and Primary Care, University of Bristol

Richard M. Martin, Professor of Clinical Epidemiology, University of Bristol

Anthony (Tony) Moore, Professor of Ophthalmology, University College London

Laurence Moore, Professor. Director of the MRC/ CSO Social & Public Health., University of Glasgow

Irwin Nazareth, Professor of Primary Care and Population Science, University College London

Goran Nenadic, Professor of Computer Science, University of Manchester

Shantini Paranjothy, Professor of Preventive Medicine Cardiff, University

Max Parmar, Professor of Medical Statistics and Epidemiology, University College London

Richard Pebody, Consultant Epidemiologist Public Health England

Steffen Petersen, Professor of Cardiovascular Medicine, Queen Mary University of London

Irene Petersen, Professor of Epidemiology and Health Informatics, University College London

Deenan Pillay, Professor of Virology, University College London

David Preen, Professor. Chair in Public Health., University of Western Australia

Kate Pickett, Professor of Epidemiology, University of York

Kathy Pritchard-Jones, Professor of Paediatric Oncology, University College London

Natasa Przulj, Professor of Biomedical Data Science, University College London

Andrew Renehan, Professor of Cancer Studies and Surgery, University of Manchester

Stephen Roberts, Reader, Swansea University

John Robson, Clinical Reader Queen Mary University of London

Sarah Rodgers, Professor of Health Informatics, Public Health and Policy, University of Liverpool, and Honorary Professor, Swansea University

Martin Rossor, Professor of Clinical Neurology, Dept of Neurodegeneration, UCL Queen Square Institute of Neurology, University College London

Ian Russell, Emeritus Professor of Clinical Trials, Swansea University

John Shawe-Taylor, Professor of Computational Statistics and Machine Learning, University College London

Aziz Sheikh, Chair of Primary Care Research and Development, University of Edinburgh

Stefan Siebert, Senior Lecturer, Swansea University

Helen Snooks, Professor of Health Services Research, Swansea University

Matthew Sperrin, Senior Lecturer, University of Manchester

Judith Stephenson, Professor of Reproductive and Sexual Health, University College London

Frank Sullivan, Professor of Primary Care Medicine, The University of St Andrews

Chris Taylor, Professor of Medical Biophysics, Professor of Computer Science, University of Manchester

Paul Taylor, Professor of Health Informatics, University College London

Adam Timmis, Professor of Clinical Cardiology Queen Mary University of London

Hester J T Ward, Consultant in Public Health Medicine NHS National Services Scotland

John Williams, Professor of Health Services Research, Swansea University

Paula Williamson, Professor of Medical Statistics, University of Liverpool

Alan Wilson, Professor of Urban and Regional Systems, University College London

Olivia Wu, Professor of Health Economics, University of Glasgow

Appointments of researcher, technical specialist and professional support staff, leveraging the Farr Institute award (*approximately 25 WTE posts over 5 years)*

Mhairi Aitken, Research Fellow, University of Edinburgh

Ashley Akbari, Senior Research Analyst, Swansea University

Mohammad Al Sallakh, Research Officer, Swansea University

Sarah Al-Adely, Data and Technical Communications Officer, University of Manchester

Samantha Alvarez-Madrazo, Research Associate, University of Strathclyde

Leslie Anne, Research Fellow, University of Edinburgh

Lorna Aucott, Senior Research Fellow, University of Aberdeen

Rowena Bailey, Research Data Analyst, Swansea University

Panos Balatsoukas, Postdoctoral Researcher, University of Manchester

Amrita Bandyopadhyay, Research Data Analyst, Swansea University

Mike Bartlett, Project Coordinator, University of Manchester

Katherine Barutcu, Research Officer, Swansea University

Catherine Batchelder, Public Engagement Officer, Swansea University

Denise Beales, Centre Manager, University College London

Miguel Belmonte, Researcher, University of Manchester

Brian Blower, System Administrator, University of Manchester

Liam Brierley, Biostatistician/Epidemiologist, University of Edinburgh

Andrew Broadbent, Software Engineer eLab Projects, University of Manchester

Paul Burton, Professor of Data Science for Health, Newcastle University

Mathilde Castagnet, Data Officer, Swansea University

Daniel Cave, Software Engineer, University of Manchester

Giovanna Ceroni, Executive Assistant to Centre Director, University College London

Tom Clemens, Research Fellow, University of Edinburgh

Huw Collins, Research Data Analyst, Swansea University

Ed Conley, Chief Scientific Officer, Farr Institute of Health Informatics Research Network, University of Edinburgh

Carol-Ann Costello, Business Relationship Manager, University of Manchester

Phil Couch, Information Systems Programme Manager, University of Manchester

Lynsey Cross, Public Engagement Officer, Swansea University

James Cunningham, Researcher, University of Manchester

Marina Daskalopoulou, Research Assistant, University College London

Emma Davies, Information and Security System Manager, University of Manchester

Joanne Demmler, Research Assistant/Officer/Lecturer in Health Informatics, Swansea University

Emma Dixon, Executive Assistant to John Ainsworth, University of Manchester

Christine Dobbs, Researcher, Swansea University

Samuel Dredge, Research Assistant, Swansea University

Vit Drga, Research Associate, University College London

Hannah Evans, Research Associate, University College London

Rachel Evans, Industry Engagement Manager, Farr Institute of Health Informatics Research Network, University of Edinburgh

Bassam Farran, Biostatistician/Bioinformatist/Machine Learning Specialist, University of Edinburgh

Natalie Fitzpatrick, Data Science Facilitator, University College London

Michael Fleming, UKRI/Rutherford Fund Research Fellow, University of Glasgow

Beata Fonferko-Shadrach, Neurology Data Research Officer, Swansea University

Sarah Fox, Public Engagement and Involvement Research Officer, University of Manchester

Richard Fry, Senior Research Officer, Swansea University

Catharine Goddard, Manager, Farr Institute of Health Informatics Research Network, University of Dundee

Arturo Gonzalez-Izquierdo, Senior Research Associate, University College London

Sharon Gordon, Programme Co-ordinator, University of Aberdeen

Ben Green, Trusted Research Environment Operations Manager, University of Manchester

Benjamin Green, Business Analyst, University of Manchester

Nick Gresham, Researcher, University of Manchester

Lucy Griffiths, Senior Research Officer, Swansea University

Rhiannon Griffiths, Programme Officer – Data Science, Swansea University

Rowena Griffiths, Research Officer, Swansea University

Kathy Haigh-Hutchinson, Software Engineer , University of Manchester

Melanie-Jayne Hainke, Learning Technologist, Swansea University

Phil Hannaford, Vice-Principal for Digital Transformation, University of Aberdeen

Douglas Hardy, Software Project Manager, University of Dundee

Steven Harris, ICT Developer, Swansea University

Nina Hayes-Thompson, Digital Communications and Events Intern, University of Manchester

Martin Heaven, Senior Research Manager, Swansea University

Neil Hillen, Data Analyst, University of Glasgow

Kate Holmes, Communications and Public Engagement Coordinator, University of Manchester

Elizabeth Irvine, Project Assistant (WARN/PAS), Swansea University

Guy Jackson, Software Engineer, University of Manchester

Nadia Jackson, Centre Manager, University College London

Emily Jefferson, Director of Health Informatics Centre, University of Dundee

David Jenkins, Researcher, University of Manchester

Camille Johnson, Project Manager, University of Manchester

Jennifer Johnston, Farr Scotland JenniferCentre Manager, University of Dundee

Michail Katsoulis, Research Associate, University College London

Daisy Kirkwood, Centre Manager, University College London

Holger Kunz, Teaching Fellow, University College London

Sabine Kurz, Communications and Event Assistant, Farr Institute of Health Informatics Research Network, University of Edinburgh

Arron Lacey, Data Analyst (PHI Unit), Swansea University

Amanda Lamb, Head of Operations, University of Manchester

Nathan Lea, Senior Research Associate, University College London

Stephen Lloyd, Software Engineer, University of Manchester

Jane Lyons, Research Data Analyst, Swansea University

Matt Machin, Information Systems Programme Manager, University of Manchester

Noel Malod-Dognin, Research Associate, University College London

Louise Marryat, Post-Doctoral Research Fellow, University of Edinburgh

Cherry Martin, Communications Manager, Farr Institute of Health Informatics Research Network, University of Edinburgh

Gordon McAllister, Software Architect, University of Dundee

Nicola McCleary, Research Fellow, University of Edinburgh

Lucy McCloughan, Scientific Development Manager, University of Edinburgh

Paul McIntosh, Farr Administrator, University of Dundee

Stephen Melia, Communications and Public Engagement Coordinator, University of Manchester

Amy Mizen, Research Officer, Swansea University

Anna Mölter, Research Associate, University of Manchester

Remi Momo, Research Associate, University College London

Alysha Morgan, CIPHER Programme Manager, Swansea University

Lynn Morrice, Centre Manager at AUKCAR, University of Edinburgh

Kate Mortimer, Centre Administrator, University of Manchester

Alireza Moayyeri, Senior Research Associate, University College London

Chris Munro, Research Associate, University of Manchester

Clifford Nangle, Database Developer/Analyst, NHS National Services Scotland

Thomas Nind, Software Architect, University of Dundee

Ruth Norris, Research Programme Manager, University of Manchester

Laura North, Prudent Healthcare Intelligence Unit Research Data Analysts, Swansea University

Kieran O'Malley, Training Officer, University of Manchester

Rhydian Owen, CIPHER Programme Manager, Swansea University

Adam Panagiotopoulos, Research Assistant, Edinburgh Law School

Vaclav Papez, Research Associate, University College London

Richard Papworth, Data Analyst, University of Glasgow
Laura Pasea, Research Assistant, University College London
Steve Pavis, eDRIS, Head of Service, Public Health and Intelligence, National Services Scotland
Jill Pell, Henry Mechan Professor of Public Health, Director, Institute of Health and Wellbeing, University of Glasgow
Julia Petschnigg, Research Associate, University College London
Carol Porteous, Patient and Public Engagement Co-ordinator, University College London
Haider Raza, M/C Learning Data Driven Analyst, Swansea University
Emma Riordan, Administrator – Farr Centre, Swansea University
Heather Robinson, Researcher , University of Manchester
Nayha Sethi, Research Fellow, Deputy Director Mason Institute, Edinburgh Law School
Aneesha Singh, Research Associate, University College London
Claire Smith, Research Projects Manager, University of Manchester
Jiao Song, Research Officer, Swansea University
Markus Steiner, Honorary Clinical Research Fellow, University of Aberdeen
Paul Stephenson, Software Developer, University of Manchester
Raj Tandon, Centre Administrator, University of Manchester
Ed Tempest, Software Engineer, University of Manchester
Rachel Thompson, Project Manager, University of Manchester
Sarah Toomey, Industrial Liaison Officer, Swansea University
Fatemeh Torabi, Research Data Analyst, Swansea University
Leandro Tramma, Senior Software Developer, University of Dundee
Sandro Tsang, Research Officer/Statistician, Swansea University
Wing-Chau Tung, Farr London Centre Manager, Farr Institute of Health Informatics Research Network Manager, University College London
Sam Turner, Research Data Analyst, Swansea University
Victoria Turner, HeRC Centre Manager, University of Manchester
Sabine Van Der Veer, Research Fellow, University of Manchester
William Vance, Software Engineer, University of Manchester
Angharad Walters, Research Assistant/Data Analyst, Swansea University
Tony Whiffen, Visiting Researcher (Welsh Government secondment), Swansea University
Andrew James Williams, Research Fellow, Scottish Collaboration for Public Health Research and Policy, Usher Institute for Population Health Sciences and Informatics, University of Edinburgh

New tenured academic appointments, leveraging the Farr Institute award

Robert Aldridge, Wellcome Trust Clinical Research Career Development Fellow and Consultant in Public Health, University College London
Samantha Alvarez-Madrazo, Research Associate, University of Strathclyde
Athanasios Anastasiou, Lecturer in Health Data Science, Swansea University Medical School, Swansea University

Amitava Banerjee, Senior Lecturer, University College London
Damon Berridge, Chair in Applied Statistics, Swansea University
Corri Black, Professor of Public Health, University of Aberdeen
Paul Burton, Professor of Data Science for Health, Newcastle University
Helen Colhoun, AXA Chair of Medical Informatics and Life Course Epidemiology, University of Edinburgh
Sarah Cunningham-Burley, Director of Centre for Biomedicine, Self and Society, Edinburgh Law School, University of Edinburgh
Juan-Pablo Casas Romero, Professor of Clinical Epidemiology and Informatics, University College London
Spiros Denaxas, Senior Lecturer, University College London
Chris Dibben, Professor of Health Geography, University of Edinburgh
Richard Dobson, Professor of Clinical Epidemiology, University College London
Michael Fleming, UKRI Research Fellow, University of Glasgow Pia Hardelid, Lecturer, University College London
Katie Harron, Senior Lecturer, University College London
Holger Kunz, Teaching Fellow, University College London
Glen Martin, Lecturer, University of Manchester
Georgina Moulton, Professor of Bio-Health Informatics Education, University of Manchester
Tanja Mueller, Research Associate, University of Strathclyde
Niels Peek, Professor of Health Informatics, University of Manchester
Clifford Nangle, Data Analyst, University of Edinburgh
Nayha Sethi, Deputy Director of Mason Institute, Edinburgh Law School
Laura Shallcross, NIHR Clinical Lecturer in Public Health Medicine, UCL Institute of Health Informatics, University College London
Matt Sperrin, Senior Lecturer, University of Manchester
Paul Taylor, Professor of Health Informatics, University College London
Sabine van der Veer, Lecturer, University of Manchester
Tjeerd van Staa, Professor of Health e-Research, University of Manchester
Alan Watkins, Associate Professor of e-trials research, Swansea University
Elizabeth Williamson, Associate Professor, London School of Hygiene & Tropical Medicine

PhD students

Adeel Waheed, Bradford Research Institute
Nida Afzal, University of Bradford
Harry Ahmed, Cardiff University
Mohammad Annas Al Sallakh, Swansea University
Adel Alhlayl, Swansea University
Victoria Allan, University College London
Haitham Alzghaibi, Swansea University
Phil Appleby, University of Dundee
Ellie Badrick, University of Manchester

Kerry Bailey, Swansea University

Natalie Berry, University of Manchester

Birgitta Bodegraven, University of Manchester

Helen Brierley, University of Manchester

Benjamin Brown, University of Manchester

Giorgio Ciminata, University of Glasgow

Rosie Cornish, University of Bristol

Eilidh Cowan, University of Edinburgh

Mattea Deliu, University of Manchester

Catherine Fitton, University of Aberdeen

Michael Fleming, University of Glasgow

Kenny Haining, University of Edinburgh

Kathryn Halliday, University of Edinburgh

Elsie Horne, University of Edinburgh

Rebecca Howard, University of Manchester

Will Hulme, University of Manchester

Myrto Kremyda-Vlachou, University College London

Emily Marchant, Swansea University

Glen Martin, University of Manchester

Tanja Mueller, University of Strathclyde

Jenny Newman, University of Liverpool

Julie Peconi, Swansea University

Tra My Pham, University College London

Rachel Reeves, University College London

Daniel Rhodes, Queen Mary University of London

William Rudgard, London School of Hygiene & Tropical Medicine

Catherine Smith, University College London

Georgious Spithourakis, University College London

Dennis Stallone Valentine, University College London

Grant Wyper, University of Strathclyde

Bilal Yassine, University College London

Anna Zylbersztejn, University College London

Fellowships funded as a result of Farr association

Robert Aldridge, Wellcome Trust Clinical Research Career Development Fellowship, University College London

Amaya Azcoaga-Lorenzo, MRC–UKRI/Rutherford Fund Fellowship at Health Data Research UK (HDR UK), University of St Andrews

Ruth Blackburn, MRC–UKRI/Rutherford Fund Fellowship at Health Data Research UK (HDR UK), University College London

Ben Brown, Wellcome Trust Research Training Fellowship, University of Manchester
Marco Caminati, MRC–UKRI/Rutherford Fund Fellowship at Health Data Research UK (HDR UK), University of St Andrews
Raymond Carragher, MRC–UKRI/Rutherford Fund Fellowship at Health Data Research UK (HDR UK), University of Strathclyde
Sheng-Chia Chung, MRC Studentship, University College London
Caroline Dale, Wellcome Trust Springboard Fellowship, University College London
Spiros Denaxas, UCL Provosts Strategic Development Fund Fellowship, University College London
Chantal Edge, NIHR Clinical Doctoral Research Fellowship, University College London
Ghazaleh Fatemifar, American Heart Association Institutional Data Fellowship, University College London
Mike Fleming (ex-Farr student), MRC–UKRI/Rutherford Fund Fellowship at Health Data Research UK (HDR UK), University of Glasgow
Julie George, Health Education England NIHR Clinical Lectureship, University College London
Alison Hale, MRC Skills Development Fellowship, Lancaster University
Michail Katsoulis, British Heart Foundation Intermediate Postdoctoral Basic Science Research Fellowship, University College London
William Harvey, EPSRC National Productivity Investment Fund Fellowship, University of Glasgow
Lamiece Hassan, MRC–UKRI/Rutherford Fund Fellowship at Health Data Research UK (HDR UK), University of Manchester
Watjana Lilaonitkul, MRC–UKRI/Rutherford Fund Fellowship at Health Data Research UK (HDR UK), University College London
Claudia Lindner, NPIF Fellowship, University of Manchester Serena Luchenski, HEE/NIHR Clinical Doctoral Research Fellowship, University College London
Tom Lumbers, MRC–UKRI/Rutherford Fund Fellowship at Health Data Research UK (HDR UK), University College London
Claire Niedzwiedz, EPSRC National Productivity Investment Fund Fellowship, University of Glasgow
Meena Rafiq, NIHR In-Practice Fellowship, University College London
Kristiina Rannikmae, UKRI Fellowship, University of Edinburgh
Laura Shallcross, NIHR Clinician Scientist Fellowship, University College London
Keith Smith, MRC–UKRI/Rutherford Fund Fellowship at Health Data Research UK (HDR UK), University of Edinburgh
Rona Strawbridge, MRC–UKRI/Rutherford Fund Fellowship at Health Data Research UK (HDR UK), University of Glasgow
Charlotte Warren-Gash, Wellcome Trust Intermediate Clinical Fellowship, University College London
Honghan Wu, MRC–UKRI/Rutherford Fund Fellowship at Health Data Research UK (HDR UK), University of Edinburgh

Farr Working Groups

e-infrastructure

Professor John Ainsworth (Chair), Professor of Health Informatics, University of Manchester

Dr Philip Couch, Information Systems Programme Manager, University of Manchester

Professor David Ford, Deputy Centre Director, and Professor of Health Informatics, Swansea University

Simon Thompson, Chief Technology Officer, Swansea University

Dr Jacky Pallas, Director, UCL Research Platforms, University College London

Professor Mark Parsons, Director of EPCC and Associate Dean for e-Research, University of Edinburgh

Dr Emily Jefferson, Director of Health Informatics Centre Services (HIC) & Co-Director of the Farr Institute of Medical Informatics, University of Dundee

Professor James Cunnigham, Professor of Strategic Management at Newcastle Business School, Newcastle University

Health Informatics Capacity Building

Professor Georgina Moulton (Chair), Professor of Bio-Health Informatics Education, University of Manchester

Athanasios Anastasiou, Lecturer in Health Data Science, Swansea University Medical School, Swansea University

Professor Paul Taylor, Professor of Health Informatics, University College London

Dr Wing-Chau Tung, Manager, Farr Institute of Health Informatics Research Network ,University College London

Dr Catharine Goddard, Manager, Farr Institute of Health Informatics Research Network, University of Dundee

Professor Colin McCowan, Professor of Health Informatics (Robertson Centre for Biostatistics), University of Glasgow

Dr Shang-Ming Zhou, Senior Lecturer in Health Informatics, Swansea University

Innovative Governance

Dr Kerina Jones (Chair), Associate Professor of Health Informatics at the College of Medicine, Swansea University

Professor Graeme Laurie, Professor of Medical Jurisprudence, University of Edinburgh

Professor James Cunningham, Professor of Strategic Management at Newcastle Business School, Northumbria University

Dr Nathan Lea, Senior Research Associate, University College London

Partnerships

Professor Harry Hemingway, (Chair), Professor of Clinical Epidemiology – Centre Director, University College London

Ms Rachel Evans, Industry Engagement Manager, Farr Institute of Health Informatics Research Network, University of Edinburgh

Professor David Ford, Deputy Centre Director – Professor of Health Informatics, Swansea University

Ms Ruth Norris, Head of Strategic Relations, University of Manchester

Ms Natalie Fitzpatrick, Data Science Facilitator and Public Engagement Coordinator, University College London

Public & Patient Involvement Engagement

Professor Sarah Cunningham-Burley (Chair), Professor of Medical and Family Sociology / Dean of Molecular, Genetic and Population Health Sciences, University of Edinburgh

Dr Mhairi Aitken, Research Fellow, University of Edinburgh

Dr Chris Carrigan, Senior Research Fellow, University of Leeds

Dr Lynsey Cross, Public Engagement Officer, Swansea University

Dr Simon Denegri, NIHR National Director for Patients, Carers and the Public, University College London

Ms Natalie Fitzpatrick, Data Science Facilitator and Public Engagement Coordinator, University College London

Dr Sarah Fox, Public Engagement and Involvement Research Officer, University of Manchester

Dr Lamiece Hassan, Public Engagement & Involvement Manager, University of Manchester

Ms Carol Porteous, Research Fellow/ PhD Student, University of Edinburgh

Dr Mary Tully, Reader in Pharmacy Practice in the Division of Pharmacy and Optometry, University of Manchester

Health Informatics Methodology Research

Iain Buchan, Professor of Health Informatics, North England, University of Liverpool

Jeremy Wyatt, Professor of Robotics and Artificial Intelligence, University of Birmingham

Cohort Study Enhancement

Ronan Lyons, Clinical Professor of Public Health Research, Director HDRUK Wales/Northern Ireland, Swansea University

Michaela Benzeval, Professor of Longitudinal Research, University of Essex

Primary Care

Brendan Delaney,Chair in Medical Informatics and Decision Making, Imperial College London

Frank Sullivan,Professor of Primary Care Medicine, The University of St Andrews

Farr Institute of Health Informatics Centre and Network Managers

Dr Alysha Morgan, CIPHER Programme Manager, Swansea University

Dr Rhydian Owen, CIPHER Programme Manager, Swansea University

Dr Denise Beales, Farr London Centre Manager, University College London

Ms Nadia Jackson, Farr London Centre Manager, University College London

Ms Daisy Kirkwood, Farr London Centre Manager, University College London

Dr Wing-Chau Tung, Farr London Centre Manager, Farr Institute of Health Informatics Research Network Manager, University College London

Dr Catharine Goddard, Manager, Farr Institute of Health Informatics Research Network, University of Dundee

Dr Lucy McCloughan, Scientific Development Manager, University of Edinburgh

Ms Jennifer Johnston, Farr Scotland Centre Manager, University of Dundee

Dr Amanda Lamb, Head of Operations, University of Manchester

Ms Ruth Norris, Research Programme Manager, University of Manchester

Ms Vicky Turner, HeRC Centre Manager, University of Manchester
